# Rab‐mediated trafficking in the secondary cells of Drosophila male accessory glands and its role in fecundity

**DOI:** 10.1111/tra.12622

**Published:** 2018-12-26

**Authors:** Elodie Prince, Benjamin Kroeger, Dragan Gligorov, Clive Wilson, Suzanne Eaton, François Karch, Marko Brankatschk, Robert K. Maeda

**Affiliations:** ^1^ Department of Genetics and Evolution, Section of Biology Sciences Faculty, University of Geneva Geneva Switzerland; ^2^ Department of Physiology, Anatomy and Genetics University of Oxford Oxford UK; ^3^ Biotechnology Center of the TU Dresden Dresden Germany; ^4^ Max Planck Institute of Molecular Cell Biology and Genetics Dresden Germany

**Keywords:** confocal microscopy, Drosophila melanogaster, male accessory glands, postmating response, Rab19, Rab6, vacuole‐like compartments

## Abstract

The male seminal fluid contains factors that affect female post‐mating behavior and physiology. In *Drosophila,* most of these factors are secreted by the two epithelial cell types that make up the male accessory gland: the main and secondary cells. Although secondary cells represent only ~4% of the cells of the accessory gland, their contribution to the male seminal fluid is essential for sustaining the female post‐mating response. To better understand the function of the secondary cells, we investigated their molecular organization, particularly with respect to the intracellular membrane transport machinery. We determined that large vacuole‐like structures found in the secondary cells are trafficking hubs labeled by Rab6, 7, 11 and 19. Furthermore, these organelles require Rab6 for their formation and many are essential in the process of creating the long‐term postmating behavior of females. In order to better serve the intracellular membrane and protein trafficking communities, we have created a searchable, online, open‐access imaging resource to display our complete findings regarding Rab localization in the accessory gland.

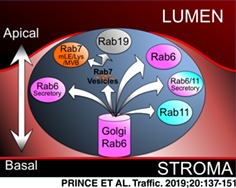

## 1 INTRODUCTION

Due to limited resources, sexual reproduction often leads to males having to compete to produce offspring in the succeeding generation.^1^, ^2^, ^3^, ^4^, ^5^ Thus, many organisms have developed methods to ensure the propagation of an individual's genome at the expense of rivals.^6^ For example, male polar bears often kill the offspring of rival males in order to favor the propagation of their own offspring.^7^ In *Drosophila melanogaster*, a more indirect “mate‐guarding” strategy is used. The seminal fluid (SF) of *Drosophila* males contains factors, called seminal fluid proteins (SFPs), which are deposited into the female during mating.^8^, ^9^ Some of these factors influence the physiology and behavior of mated females to favor the reproductive success of the mating male.^8^, ^9^, ^10^ The male‐induced changes in mated females are called the postmating response (PMR). Some characteristics of the PMR are: (1) a decrease in mating receptivity,^11^, ^12^ (2) a reduction of female life span,^13^ (3) the storage of sperm,^14^, ^15^, ^16^ (4) an increase in ovulation,^17^, ^18^ (5) a modification in feeding behavior^19^ and (6) a remodeling of the gut.^20^ Although similar strategies have also been described for mammals, like changes in ovulation frequency and immune responses in females after mating,^21^, ^22^ the mechanistic principles are less well understood.

While in mammals, SFPs are mostly produced in the prostate gland, the seminal vesicles and the bulbourethral gland, in *Drosophila* males, these proteins are primarily produced by a single, paired‐gland called the accessory gland (AG). The *Drosophila* AG is a two‐lobed structure, made of two types of bi‐nucleated and secretory cell types arranged in a cellular monolayer that surrounds a central lumen and is wrapped by a layer of muscle cells. The two types of secretory cells are called the main cells (MCs) and the secondary cells (SCs). The hexagonally shaped MCs make up ~96% of the secretory cells of the gland and are known to produce the vast majority of the SFPs.^23^, ^24^ The remaining 4% of secretory cells are the SCs, which are located only at the distal tip of each lobe, interspersed with MCs; they are much larger, spherically shaped cells that are filled with a number of large, vacuole‐like compartments (VLCs).^25^, ^26^, ^27^ The VLCs are membrane‐bound organelles containing a large internal space. The SCs, like the MCs, are in direct contact with the glandular lumen and are able to contribute to the seminal fluid.^25^, ^26^, ^28^, ^29^, ^30^, ^31^, ^32^ Recent findings show that the SCs, however, are not crucial for initiating PMR behaviors. Instead, through genetic manipulations that affect SCs and/or their VLCs, SCs have been shown to play a critical role in sustaining the female PMR for up to 10 days after mating.^26^, ^29^, ^30^, ^31^, ^32^ Given their prominence in SC architecture, the biological function of VLCs seems to be key to understanding how SCs function in sustaining the PMR. In mammals, similar VLCs have been implicated in different intracellular trafficking pathways such as endocytosis^33^ and secretion.^34^

Intracellular membrane and protein traffic is regulated by a family of membrane‐associated, small GTPases called Rabs (Ras‐like bovine proteins). Because Rabs control individual trafficking sub‐steps, these proteins are suitable to identify cellular membrane compartments.^35^, ^36^

Apico‐basolaterally polarized secretory cells (such as salivary gland cells, SG) often show a very specialized organization of their Rab machinery.^37^ Close to their apical membrane, these cells seem to form unusual Rab11, Rab6 and Rab30 compartments.^37^ Rab6 and Rab11 are implicated in the secretory and recycling pathway starting from the Golgi apparatus.^38^, ^39^, ^40^ The function of Rab30 is less clear. It has been suggested that Rab30 regulates Golgi‐related transport, though conclusive evidence is still missing.^41^ Structurally close to Rab30 is Rab19. As both GTPases show overlapping localization in SG cells,^37^, ^42^ it has been suggested that these proteins may be, to some extent, functionally redundant.^41^ On the other hand, in SG cells, the endocytic compartments (eg, Rab5 and Rab7 endosomes) are predominantly localized to the basolateral area of the cells (Rab7 and Rab4) or uniformly distributed within the cytoplasm (Rab 5).^37^ Therefore, it would be interesting to know if other secretory cells show a similar re‐organization of their Golgi‐related secretory machinery, and how such designs rely on the individual cell function.

Recently, a collection of YFP‐tagged *rab* knock‐in alleles was established in *Drosophila*.^37^ This Rab library allows in vivo tracking of the Rab proteins in any given cell type at their endogenous expression levels.^37^ Here, we use the Rab library to screen for the expression and localization of all *Drosophila* Rab proteins in the male AG. Focusing on the SCs, we show that Rab6, 7, 11 and 19 define four different VLC populations. This extends previous studies that showed that there were at least two different subclasses of VLCs using numerous intracellular markers.^25^, ^28^, ^29^ Furthermore, we track the development of VLCs over the first few days after male eclosion and find that the first VLCs that we detect are Rab6‐positive, while Rab7‐, Rab11‐ and Rab19‐positive compartments appear later in adulthood, suggesting they may be Rab6‐dependent. Consistent with this observation, the genetic reduction of Rab6 prevents the formation of all VLC classes. In contrast, the absence of Rab7 and 11,^29^, ^30^ but not Rab19, results in the loss of only some VLC populations. These losses, however, are sufficient to result in defective female PMR behaviors. Finally, we have established an online image reference platform (https://flyrabag.genev.unige.ch). This resource provides annotations based on a defined vocabulary for each Rab protein expressed in AGs and allows 3D localization tracking down to subcellular resolutions. Thus, our work maps out the maturation of the membrane/protein transport machinery in the AGs over time and adds valuable knowledge to the existing model describing the SC secretion system.^24^, ^25^, ^27^, ^28^, ^29^, ^30^, ^31^, ^43^

## 2 RESULTS

### 2.1 The basic morphology of the accessory gland epithelium

**Figure 1:**
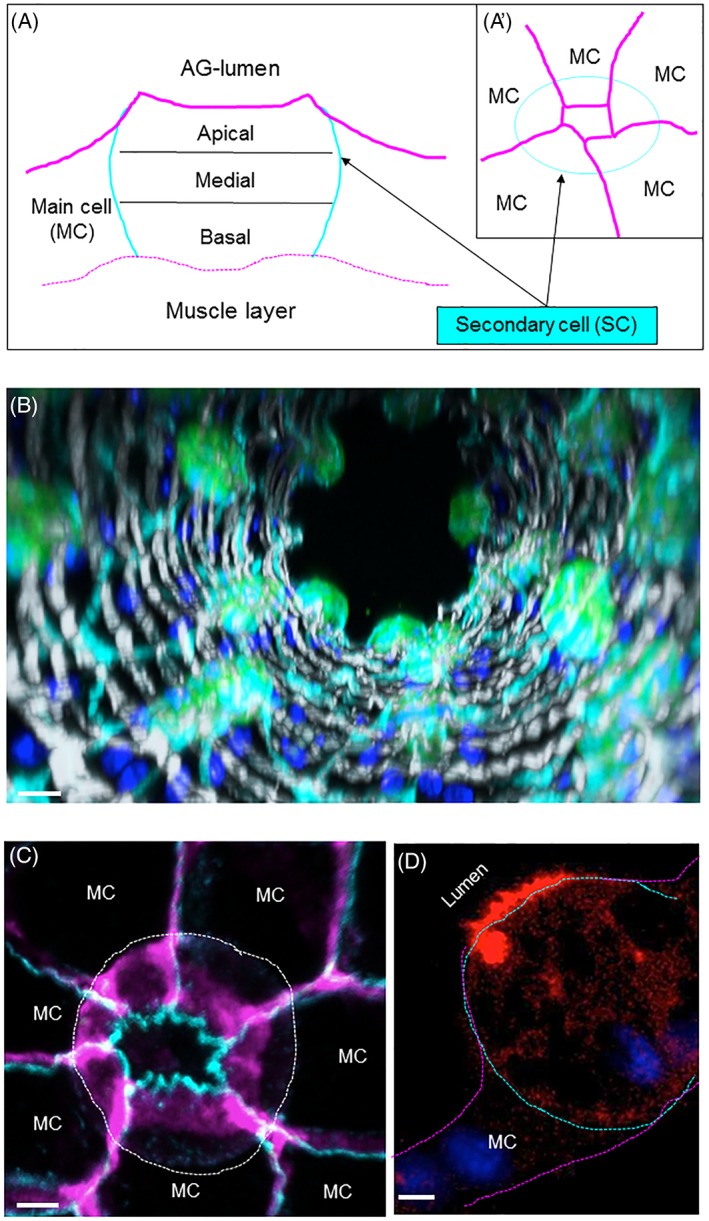
Organization of the SCs. (A‐A′) Schematic depiction of a secondary cell (SC, light blue) and flanking main cells (MC, magenta). SCs are embedded in MCs. We divided SCs into three zones: Apical, Medial and Basal (A, sagittal view). The apical, luminal contact zone of SCs is very small (A', top‐view, magenta). (B) Confocal images from the distal tip of an AG are assembled into a 3D projection down the long axis of the gland. GFP‐ (green) expressing SCs are probed for Dlg (cyan), F‐actin (light‐gray) and DAPI (dark blue). Scale bar = 15 μm. (C) Compressed confocal stack (15 μm from apical to basal) shows a SC (outlined by a white dashed line) probed for DCAD (cyan) and Dlg (magenta). Surrounding main cells (MCs) are indicated, scale bar = 5 μm. (D) A confocal slice along the apical‐basal axis of a representative SC (outlined with a dashed line) expressing the F‐actin marker, Lifeactin‐Ruby (red), and stained with DAPI (blue). The apical side of the cell is at the top left and the basal side is at the bottom right. Note the enrichment of actin filaments at the apical membrane that is facing the AG lumen. Scale bar = 5 μm

An ultrastructural study of the AGs performed by Bairati^25^ suggested that both gland cell types are polarized and secretory in nature. To confirm this notion, we examined the cellular localization of the canonical cell polarity markers, DE‐Cadherin (DCAD, marking apical adherence junctions) and Discs‐Large (Dlg, marking basolateral membrane), as well as the F‐actin staining molecules, Phalloidin and Lifeactin,^44^, ^45^ in *Drosophila* AGs. Confirming the findings of Bairati, we find that the cells of the AG are indeed polarized, with their apical surface facing the central lumen (Figure 1B,D). One striking characteristic of the cellular monolayer making up the AGs is seen in regards to the SCs. The SCs display a distinct round shape and seem to extrude out from the uniform sheet of MCs into the luminal space (Figure 1A,B). However, even with this extrusion, they do not display a large exposed surface to the luminal fluid, as the MCs surrounding the SCs seem to extend over much of their surface to restrict contact with the lumen (Figure 1A,C).^28^ This spreading of the MCs over the SCs also results in a large contact zone between the two cell types. Furthermore, we find a dense F‐actin network concentrated along the apical surface of the SCs, with actin‐filled membrane protrusions extending into the luminal space (Figure 1D).^25^ This apical enrichment of F‐actin is reminiscent of other secretory gland cells (eg, SG cells) and may reflect the secretory nature of SCs.^37^, ^46^

### 2.2 Four different Rabs mark vacuole‐like compartments in secondary cells

**Figure 2:**
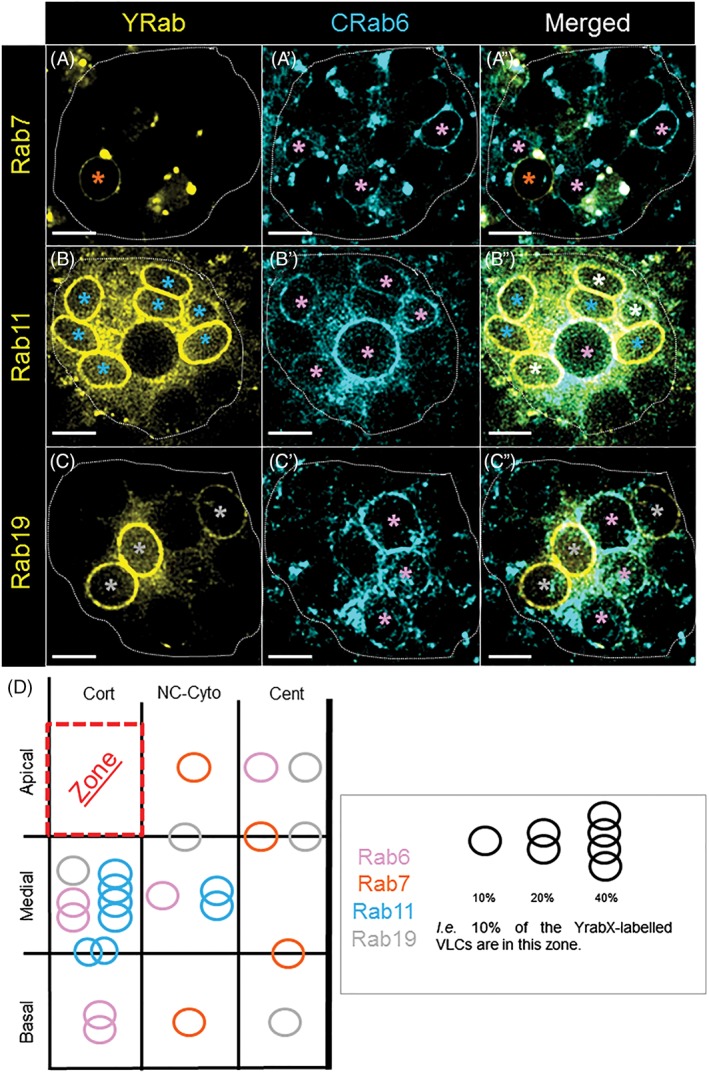
The Rabs associated with VLCs. (A‐C″) Shown are individual wide‐field florescence microscopy slices (0.8 μm slices) of SCs at three levels along the apical‐basal axis (A‐A″ Apical; B‐B″ Basal; C‐C″ Apical) from *Crab6; Yrab7*, *Crab6; Yrab11* or *Crab6; Yrab19* flies. Non‐fixed AGs were visualized for YFP (yellow; A, B, C) and CFP (cyan; A′, B′, C′) fluorescence. Asterisks indicate labeled VLCs (orange, Yrab7; pink, Crab6; light blue, Yrab11; gray, Yrab19; white, for VLCs in the merged images that co express two Rabs), scale bars = 5 μm. (D) Plotted is the relative position and abundance of VLCs labeled by one Rab in SC cells; color code and symbols are annotated in the legend (right box). One circle indicates that 10% of the YrabX‐labeled VLCs per cell are localized in this indicated zone (Rab6, *n*_cell_ = 19, *n*_VLCs_/cell = 6.37 ± 2.69; Rab7, *n*_cell_ = 8, *n*_VLCs_/cell = 4.38 ± 3.34; Rab11, *n*_cell_ = 5, *n*_VLCs_/cell = 9.4 ± 2.7; Rab19, *n*_cell_ = 13, *n*_VLCs_/cell = 6.08 ± 2.93). Cort, cortical; NC‐Cyto, noncentral cytoplasmic; Cent, central. Note that the percentages of vacuoles in a specific location are only mentioned if they reach each threshold (10% or above). Because of this, the total percentage of vacuoles do not necessarily add up to 100% in the diagram

Although VLCs are prominent in SCs,^25^, ^26^, ^27^ their molecular organization and function remains elusive. We hypothesized that VLCs could be trafficking compartments required for the efficient secretion of SFPs. To study the role of VLCs in membrane trafficking, we screened all the *Drosophila* Rab proteins using the YRab‐library^37^ (http://rablibrary.mpi-cbg.de/). To annotate the localization of each expressed Rab protein, we used a defined terminology (see Section 4) and created a CATMAID‐based website (https://flyrabag.genev.unige.ch) to present original confocal data sets of the localization patterns of each of the expressed Rab proteins in both MCs and SCs. In this way, users are able to navigate and track Rab compartments at subcellular resolution. Here, we will primarily focus on the Rab localization patterns in the SCs. Overall, we find that 16 Rabs are expressed in SCs and that 4 of these Rabs are associated with VLCs: Rab6, 7, 11 and 19 (Figure 2, https://flyrabag.genev.unige.ch).

### 2.3 Rab6 is associated with the *trans‐*Golgi network and VLCs

**Figure 3:**
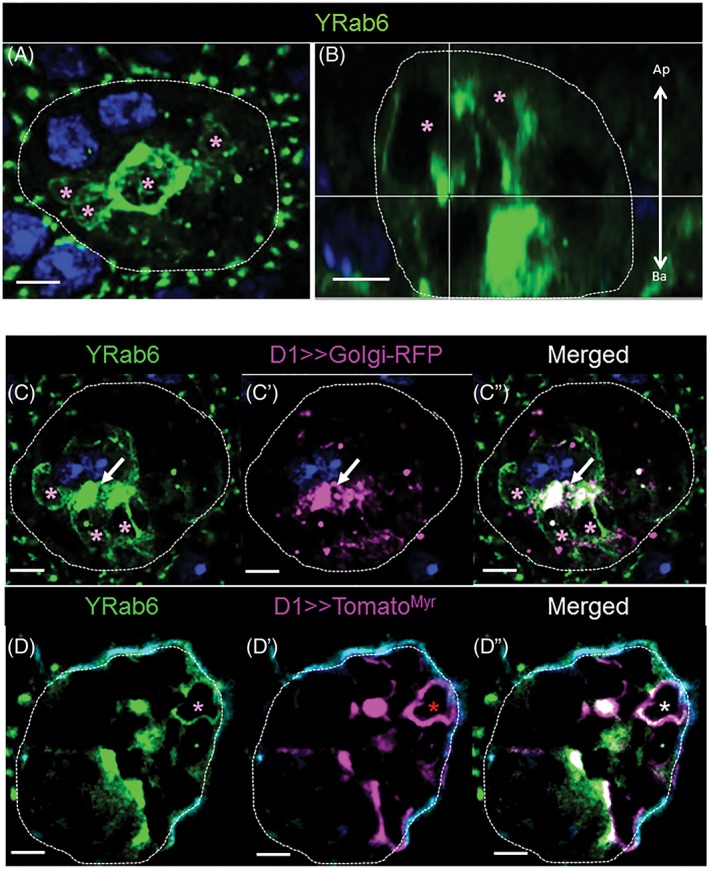
Organization of Rab6 membranes in SCs. (A, B) Shown is a Z‐projection (A, 15 μm; Medial; top‐view) and a confocal reconstruction of a sagittal section from the same confocal stack (B, 0.8 μm; sagittal‐view) of an Yrab6 SC probed for DAPI (blue) and YFP (green). Pink asterisks indicate Yrab6‐VLCs, scale bars = 5 μm, arrow shows the apical‐basal cell orientation (B). (C‐D″) Shown are confocal slices of single SCs (0.8 μm thick slices; Medial) from *Yrab6; D1> > golgiRFP* (C‐C″) and *Yrab6; D1> > tomato* ^*Myr*^ (D‐D″) flies. AGs were probed for YFP (green), DAPI (dark blue) and RFP/Tomato (magenta). Asterisks indicate labeled VLCs (pink, Yrab6; red, Tomato/RFP markers; white asterisk and arrows, for colocalization), scale bars = 5 μm

In many cell types, Rab6 is known to localize within the *trans*‐Golgi network (TGN) and to regulate protein and membrane traffic from the Golgi organelle to other membrane targets.^37^, ^38^, ^47^, ^48^ To test if VLCs are interconnected to the TGN, we probed *Yrab6* glands together with a battery of known Golgi‐markers.^37^, ^49^, ^50^ As expected, Rab6 is associated with the Golgi organelle in SCs (Figure 3C). However, the appearance of the Golgi in SCs (Figure S1A in Appendix S1, Supporting Information) is very different from the Golgi organelle in other cells (eg, MCs).^25^ In most *Drosophila* cell types, multiple Golgi units are dispersed and their build‐up is primitive, consisting of a single *cis‐*Golgi and *trans‐*Golgi membrane sheet.^48^, ^51^, ^52^, ^53^, ^54^ In SCs, we find that the Golgi forms a central, extended structure within the basal‐medial area of the cell (Figure S1A in Appendix S1).

The VLCs bound by Rab6 (*n*_cell_ = 19; *n*_VLCs_/cell = 6.37 ± 2.69), however, display no Golgi signature (Figure 3C‐C″). Rab6‐VLCs appear mainly in two areas: most are found in the basal‐to‐medial part of the cell along the plasma membrane, while other VLCs appear apically localized in the non‐central (NC)‐cytoplasmic and central regions of the cell (Figures 2D and 3A,B; Figure S2A in Appendix S1 and Section 4 for location terminology). The distribution of Rab6 in SCs is somewhat similar to SG cells, where Rab6 is found on Golgi but also on non‐Golgi compartments.^37^ Nevertheless, the extreme enlargement of all Rab6 compartments in the SCs and the more‐differentiated morphology of the Golgi, point to extensive membrane/protein transport processes.^55^, ^56^, ^57^, ^58^

The myristoylated fluorescent protein, Tomato^Myr^, associates with distinct cellular membranes including the plasma membrane, and its lipid modification prevents free intracellular diffusion.^59^ Therefore, we presume that Tomato^Myr^ needs to enter the secretory route to reach the cell periphery^44^, ^60^, ^61^ (Figures S3 and S4 in Appendix S1). To test if Rab6‐VLCs shuttle proteins along this secretory route, we expressed Tomato^Myr^ in Yrab6 SCs. Our results show that the cortical and NC‐cytoplasmic Rab6‐positive VLCs are used to transport this reporter protein (Figure 3D‐D″). The role of Rab6‐VLCs in secretion is further supported by tracking a different cell surface marker, CD8‐RFP and two known SFPs (CG1656 and CG17575) expressed in the secondary cells. In each case, these molecules are found in some Rab6‐VLCs (Figure S5A,D and S6A in Appendix S1), confirming that Rab6‐VLCs are probably used along the route for secreting proteins.

### 2.4 Rab11‐VLCs partially overlap with Rab6 traffic

**Figure 4:**
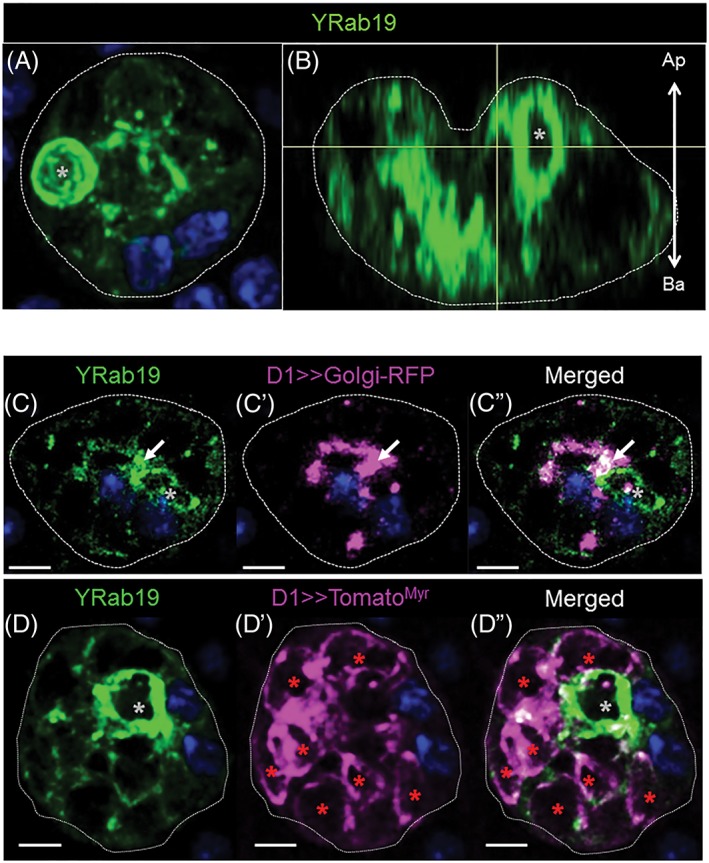
Organization of Rab11 membranes in SCs. (A, B) Shown is a Z‐projection (A, 15 μm; Medial; top‐view) and a confocal reconstruction of a sagittal section from the same confocal stack (B, 0.8 μm; sagittal‐view) of an Yrab11 SC probed for DAPI (blue) and YFP (green). Light blue asterisks indicate Yrab11‐VLCs, scale bars = 5 μm, arrow shows the apical‐basal cell orientation (B). (C‐D″′) Shown are confocal slices of single SCs (0.8 μm thick sections; Basal) from *Yrab11; D1> > golgiRFP* (C‐C″) and *Crab6*; *Yrab11, D1>>tomato* ^*myr*^ (D‐D″′) flies. AGs were probed for YFP (green), CFP (cyan) and RFP/Tomato (magenta). Asterisks indicate labeled VLCs (light blue, Yrab11; red, Tomato marker; pink, Crab6; white, for colocalization), scale bars = 5 μm. A VLC marked by the Crab6, Yrab11 and *tomato* ^*myr*^ is indicated by a white asterisk (D″′)

Rab11 is generally associated with recycling endosomes^35^, ^36^, ^62^, ^63^, ^64^ and is known to contribute to SC secretory activity.^29^, ^30^ Previously, ectopically expressed Rab11 was shown to mark VLCs containing ANCE, an enzyme later found in the lumen of the AG. This suggested that those VLCs played a role in secretion. Here, we confirm these results, finding that endogenously expressed, tagged Rab11 marks a number of VLCs, primarily located near the plasma membrane (cortical) around the basal/medial and medial part of the SCs (*n*_cell_ = 5; *n*_VLCs_/cell = 9.4 ± 2.7). Yrab11 also marks punctae, basally‐to‐medially enriched in the cytoplasmic compartment as well as in the central area with an apical enrichment (Figure 4). As Rab11 and Rab6 seem to be present on VLCs located in the same zone of the SCs (Figure 2B‐B″, Figure 2D), we tested if Rab6 and 11 colocalize to certain VLCs. To do this, we examined AGs that express both Yrab11 and Crab6 (like Yrab6 but marked with CFP). Using these tools, we find that Rab6 and Rab11 coexist on a subset of VLC membranes (Figure 2B″), suggesting that both of these VLCs may be involved in the same secretion pathway. To test this, we expressed Tomato^Myr^ in the SCs of *Crab6; Yrab11* males. Importantly, we find Tomato^Myr^ protein in CRab6‐, YRab11‐ and CRab6/YRab11‐VLCs, suggesting that Rab11‐VLCs probably participate in the same secretion pathway as Rab6 (Figure 4D‐D″′).^38^, ^39^ This is also supported by staining of the CG1656, CG17575 and CD8‐RFP, which can all be found in Rab11 and Rab6 VLCs (Figures S5 and S6 in Appendix S1). Interestingly, the staining pattern of some of the secreted markers examined (particularly CG17575) marked dense‐core structures within the VLCs, similar to that previously reported with ANCE within the Rab11 marked VLCs.^29^, ^30^, ^65^

### 2.5 Rab19‐labeled VLCs are dependent on *Rab7*

**Figure 5:**
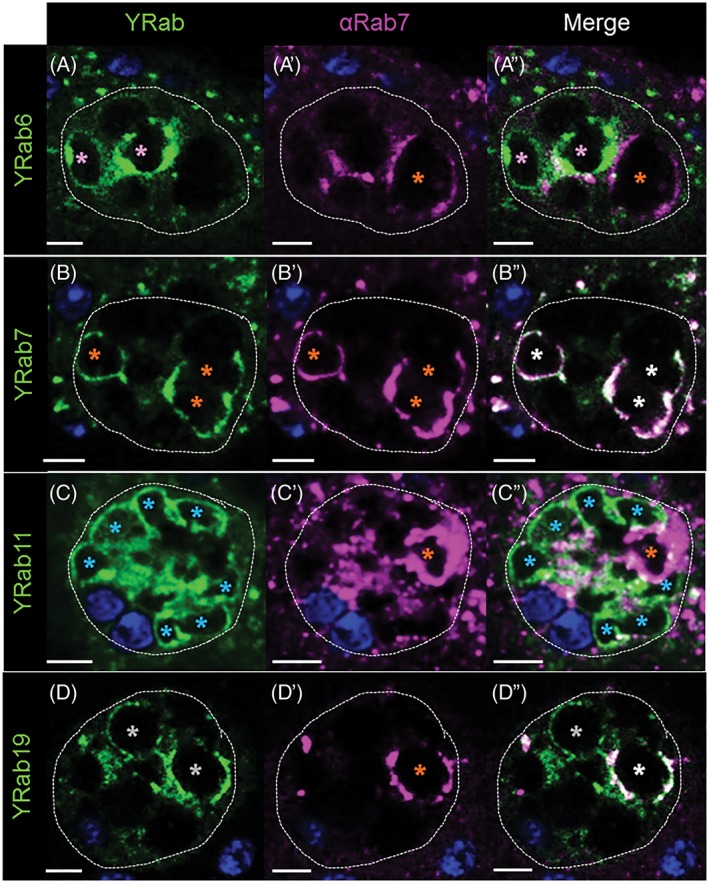
Organization of Rab19 membranes in SCs. (A, B) Shown is a Z‐projection (A, 15 μm; Medial; top‐view) and a confocal reconstruction of a sagittal section from the same confocal stack (B, 0.8 μm; sagittal‐view) of an Yrab19 SC probed for DAPI (blue) and YFP (green). Gray asterisks indicate Yrab19‐VLCs, scale bars = 5 μm, arrow shows the apical‐basal cell orientation (B). (C‐D″)Shown are confocal slices of single SCs (0.8 μm thick sections; Apical) from *Yrab19; D1> > golgiRFP* (C‐C″), and *Yrab19; D1> > tomato* ^*myr*^ (D‐D″) flies. AGs were probed for YFP (green), DAPI (dark blue) and RFP/Tomato (magenta). Asterisks indicate labeled VLCs (gray, Yrab19; red, Tomato/RFP markers; white, for colocalization), scale bars = 5 μm

In SGs, Rab19 is found exclusively associated with the apical portion of the cell.^37^ Although the biological role of Rab19 is poorly understood, because of its localization, Rab19 has been suggested to be involved in apical secretion.^37^ We found that, in AGs, Rab19 is absent in MCs, but is strongly expressed in SCs, where it is associated with VLCs mainly localized towards the apical side of the cells (Figure 5B, *n*_cell_ = 13; *n*_VLCs_/cell = 6.08 ± 2.93). Colocalization experiments with the Rab6 marker shows that Rab19‐VLCs do not colocalize with Rab6 (Figure 2C‐C″). We did find, however, that a small proportion of Tomato^Myr^ may be present in these compartments (Figure 5D‐D″ and Figure S4D‐D″ in Appendix S1) and that some Rab19 is also present on the Golgi membrane (Figure 5C‐C″). Other secretory markers, like CG1656 and CG17575, are also found in some, but not all Rab19 VLCs (Figure S5C‐C″ and S5F‐F″ in Appendix S1). Taken together, these results suggest that Rab19 VLCs may play a role in secretion.

**Figure 6:**
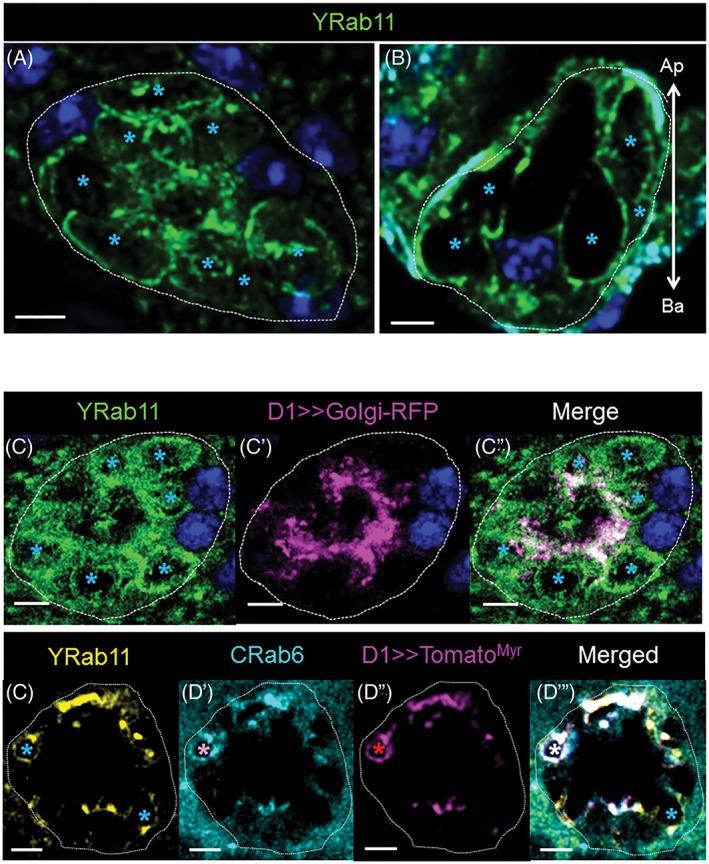
Rab19 and Rab7 colocalize to some VLCs. (A, D″) Shown are confocal intersections of single secondary cells (0.8 μm; A‐A″ Medial; B‐B″ Basal‐Medial; C‐C″ Medial; D‐D″ Medial‐Apical) from *Yrab6* (A), *Yrab7* (B), *Yrab11* (C) or *Yrab19* (D) flies. AGs were probed for YFP (green; A, B, C, D), for anti‐Rab7 (magenta; A′, B′, C′, D′) and for DAPI (dark blue). Asterisks indicate VLCs (pink, Yrab6; orange, Yrab7 and anti‐Rab7; light‐blue, Yrab11; gray, Yrab19; white on the merged pictures for VLCs that co‐express YFP and Rab7), scale bars = 7 μm

**Figure 7:**
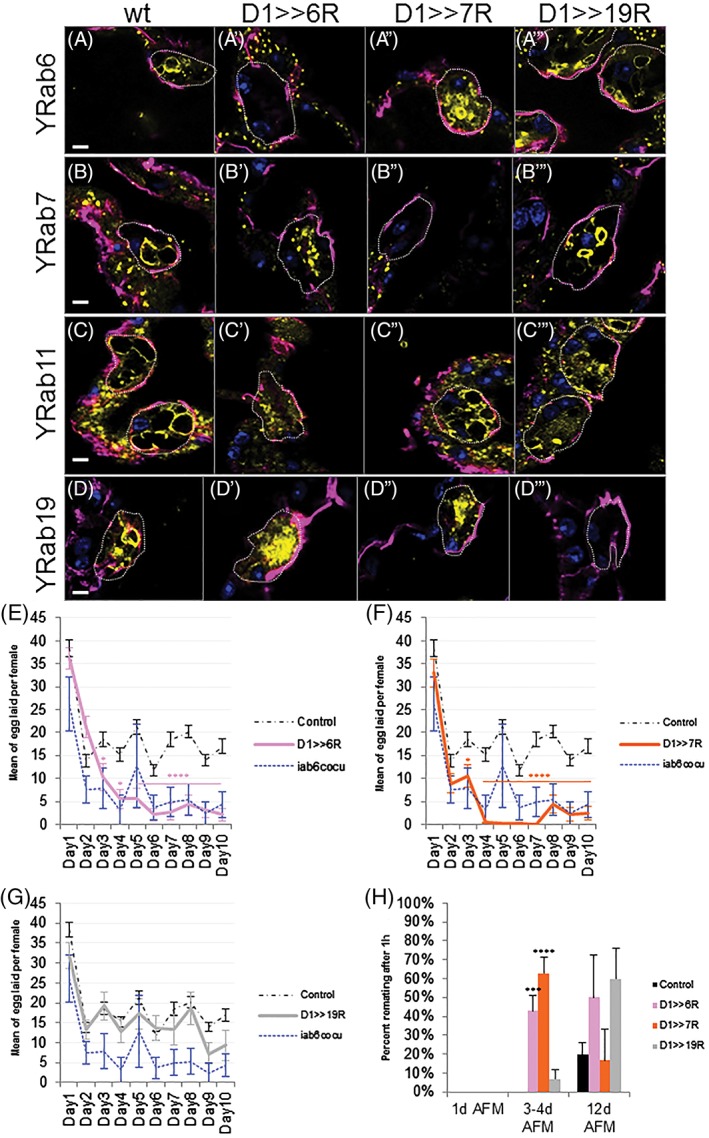
Rab6 is instructive for VLC formation and the female PMR. (A‐D″′) Shown are confocal slices (0.8 μm thick sections; top‐view; A‐A″′, Medial; B‐B″′ and D‐D″′, Apical; C‐C″′, Basal) of SCs from *Yrab6* (A)*, Yrab6;D1> > rab6* ^*RNAi*^ (A′)*, Yrab6;D1> > rab7* ^*RNAi*^ (A″)*, Yrab6;D1> > rab19* ^*RNAi*^ (A″′)*, Yrab7 (B), Yrab7;D1> > rab6* ^*RNAi*^ (B′)*, Yrab7;D1> > rab7* ^*RNAi*^ (B″)*, Yrab7;D1> > rab19* ^*RNAi*^ (B″′)*,Yrab11* (C)*, Yrab11;D1> > rab6* ^*RNAi*^ (C′)*, Yrab11;D1> > rab7* ^*RNAi*^ (C″)*, Yrab11;D1> > rab19* ^*RNAi*^ (C″′)*, Yrab19* (D)*, Yrab19;D1> > rab6* ^*RNAi*^ (D′)*, Yrab19;D1> > rab7* ^*RNAi*^ (D″) and *Yrab19;D1> > rab19* ^*RNAi*^ (D″′) *flies*. AGs were probed for YFP (yellow), Dlg (magenta) and DAPI (blue). Scale bars = 5 μm. (E‐G) Plotted are the number of eggs laid over a 10‐day period for *wild‐type* females mated with males of following genotypes: *D1‐Gal4* and *Canton‐S* (control, black broken line, *n* = 84), *iab6* ^*cocu*^ (blue broken line, *n* = 6), *D1> > rab6* ^*RNAi*^ (E, pink, *n* = 19), *D1> > rab7* ^*RNAi*^ (F, orange, *n* = 19), *D1> > rab19* ^*RNAi*^ (G, gray, *n* = 14). SE of the mean is indicated. Statistics, *, *P* < 0.05; **, *P* < 0.01; ****P < 0.00001; Mann‐Whitney *U* test. (H) Shown is the re‐mating frequency (in %) of *wild‐type* females, previously mated with males of following genotypes: *D1> > GFP* (control, black, *n* = 67), *D1> > rab6* ^*RNAi*^ (pink, *n* = 17), *D1> > rab7* ^*RNAi*^ (orange, *n* = 17), *D1> > rab19* ^*RNAi*^ (gray, *n* = 16). The males used for the secondary matings are *wild‐type*. AFM indicates time after the initial mating, error bars indicate SE of the mean. Statistics, *, *P* < 0.05; **, *P* < 0.01; ****P < 0.00001; Mann‐Whitney *U* test

Rab7 is a molecule known to regulate late endosomal traffic and is enriched on lysosomes.^29^, ^66^, ^67^ In addition, Rab7 has often been found on ER exit sites where it co‐localizes with Rab1.^68^ The association of Rab7 with ER/*cis*‐Golgi membranes may indicate the formation of specialized Rab7 compartments that enter non‐canonical trafficking routes.^69^ Previous results have shown that Rab7 sometimes colocalizes with Rab11 on SC VLCs, which has been suggested as a link between multi‐vesicular body formation and secretion.^29^ In the SCs, we observe that Rab7 is associated with VLCs that are Rab6‐negative (*n*_cell_ = 8; *n*_VLCs_/cell = 4.38 ± 3.34) (Figures 2A‐A″ and 6A‐A″). Surprisingly, however, we found that Rab7 sometimes colocalized with Rab19 on VLCs (Figure 6D‐D″). We thus speculated that Rab7 membranes might have a relationship to Rab19‐VLCs. To test our idea, we knocked down *rab7*. Consistent with this prediction, loss of Rab7 results in the complete depletion of Rab19‐ (Figure 7D″; Figure S9 in Appendix S1) but not Rab6‐VLCs (Figure 7A″; Figure S9 in Appendix S1). Depletion of Rab19 also showed changes in the appearance of Rab7‐VLCs but is not required for Rab7‐VLC creation (Figure 7B″′; Figure S9 in Appendix S1) (also, see below). Thus, there is a clear connection between Rab7 and Rab19‐VLCs. Previous data has shown that there were at least two types of Rab7 compartments in SCs, based on the staining of the pH sensitive dye, lysotracker.^29^ As none of the Rab19‐positive VLCs stain with lysotracker, the Rab7/Rab19‐positive VLCs must be non‐acidic compartments (Figure S7B‐B″ in Appendix S1), similar to VLCs previously described that we found to be Rab7‐ and Rab11‐positive.^29^

### 2.6 VLC formation and the female long‐term PMR are Rab6‐dependent

Recent studies have shown that males require 4 days to reach full sexual potency, based on the PMR.^70^ To investigate if VLCs change their molecular identity in that developmental timeframe, we tracked VLC formation in SCs over time (Figure S8 in Appendix S1). Interestingly, 1 hour after eclosion, SCs show exclusively Rab6‐VLCs. Later, at 4 hours post‐eclosion, Rab19‐ and Rab11‐VLCs become visible. It is only after 3 days that Rab7‐VLCs appear, though smaller Rab7 compartments are visible at eclosion. Over the next 2 days, the VLCs continue to grow in size and number until 5 days post‐eclosion, when the cells seem to reach a stable structure. These results, taken together with the functional data of others,^70^ correlate the development of the VLCs with AG functionality in newly eclosed adult males.^70^

The association of specific Rabs to maturing VLCs begs the question of whether or not the Rabs are directly required for the formation of the VLCs and SC functionality. Previously, we have shown that the maintenance of the PMR is impaired in mutants that lack all VLCs.^26^, ^71^ Here, we chose to individually knock‐down each candidate *rab* in SCs and to assay VLC appearance and the long‐term, female PMR. Strikingly, knocking down *rab6* in the SCs leads to the disappearance of all VLCs in mature AGs (Figure 7A′‐D′; Figure S9 in Appendix S1). It is interesting to note that smaller vesicles marked by Rab7, Rab11 and Rab19 are still present (Figure 7B′‐D′) and that the Golgi‐RFP marker still marks elements of a central channel (Figure S11D in Appendix S1). Thus, the loss of VLCs in the *rab6* knock‐down cannot simply be explained by either the absence of the Rab7, 11 or 19 proteins (Figure 7B′‐D′; Figure S9 in Appendix S1), or the complete lack of Golgi apparatus (Figure S11C,D in Appendix S1) and may thus be the result of a Rab6‐VLC‐dependent maturation process.

The loss of Rab6 in SCs also results in a dramatic decrease in the long‐term but not short‐term PMR (Figure 7E,H). Although the PMR starts normally, the mating‐induced egg‐laying stimulation (Figure 7E) and the unreceptiveness for a secondary mating (Figure 7H) is not sustained past the first 2 days post‐mating. This is similar to the PMR seen in mates of *iab6* ^*cocu*^ mutant males who similarly also lack all VLCs in the SCs (Figure 7E).^26^, ^71^ Although the systematic functional analysis of *rab11* was prevented due to general lethality using our driver, we were able to test for the effects of *rab7* and *rab19* knock‐down. The depletion of either *rab* did not affect the formation of Rab6‐ (Figure 7A′ and 7D′) or Rab11‐VLCs (Figures 7A″′ and 7D″′). However, the absence of Rab7 in SCs prevents the formation of Rab19‐VLCs and also changes the long‐term PMR (Figure 7F,H). As the knockdown of *Rab19* does not affect the long‐term PMR, we presume, that the effect of *rab7* depletion on the PMR stems from a central endocytic block that impairs the general functionality of SCs.^29^ Following this interpretation, Rab19 may belong to an unrelated, more specialized trafficking pathway or simply be redundant with another secretion pathway.

## 3 DISCUSSION

Recent findings regarding the principles of intracellular protein/membrane trafficking have shown that different cell types show a surprising versatility with regards to their usage of the intracellular transport machinery. Although the Rab protein family regulates intracellular trafficking steps across various cell classes (ie, epithelia), the composition, organization and trafficking function of Rab proteins often seems different.^37^, ^68^, ^72^ Therefore, it is vital to chart and understand intracellular trafficking pathways in as many suitable model systems as possible to describe general transport principles, like continuous or pulsed secretion.^30^, ^37^, ^38^, ^68^, ^72^

Here, we describe the organization of the protein trafficking pathway in the SCs of male *Drosophila* AGs and lay down a molecular foundation for Rab‐dependent transport routes in this cell type. We and others found that SCs are embedded in a monolayer of primary cells (MCs) and that their apical side faces the central gland lumen.^25^, ^28^, ^29^, ^30^ However, it is interesting to note that the luminal membrane of SCs is highly restricted by the surrounding MCs and that there is a large apical SC/MC contact zone. These overlapping membranes may form an intercellular cavity and secreted proteins could be captured between apical adherence and baso‐lateral contact zones. Such a morphological feature could facilitate paracellular transport.^73^, ^74^, ^75^ If true, SCs are perfectly positioned to receive material from neighboring MCs (or vice versa) and to secrete these products into the gland lumen. In support of this idea, it was reported that the SFP, Ovulin, is produced in MCs but is found in the SC VLCs.^24^, ^26^ This finding implies that MC‐produced Ovulin can be endocytosed by SCs for protein modification.^26^

To begin to investigate how these cells function, we decided to describe the transport machinery of SCs with our main focus on the Rab proteins and the Golgi network. We found that most Rabs are expressed in SCs and we used a defined terminology to annotate their intracellular localization. Our data are presented in our open access online platform (https://flyrabag.genev.unige.ch) and the approach is complementary to an already published online resource for other *Drosophila* cell types^37^ (http://rablibrary.mpi-cbg.de/).

One predominant intracellular compartment in SCs is the VLC.^25^, ^26^, ^27^ These membrane compartments are known to be critical for SC function^26^, ^29^, ^30^ and are suggested to be involved in the secretory transport route.^26^, ^29^, ^30^ Indeed, Bairati and others have presented evidence indicating that these structures occasionally fuse with the plasma membrane to release their cargo.^25^, ^29^, ^30^ We found that, among the Rab proteins, only Rab 6, 7, 11 and 19 are associated with VLCs. Interestingly, these Rabs define distinct populations of VLCs and form after a maturation process that correlates with the time AGs need to assume their optimal biological functionality.^70^

Rab6 is a well‐studied core Rab protein^38^, ^47^, ^48^, ^76^, ^77^ associated with the TGN,^76^, ^78^ reported to regulate retrograde traffic from the Golgi to the ER^79^, ^80^ and transportation of cargo destined for secretion.^38^, ^48^, ^81^ In SCs, we found Rab6 associated with the Golgi network as well as a subset of non‐Golgi VLCs. The Golgi of the SCs forms an extended central structure, which is unusual for *Drosophila* cells. Most cell types in *Drosophila* possess many solitary Golgi organelles that consist of one *cis*‐Golgi and one *trans*‐Golgi membrane sheet.^51^, ^52^, ^53^, ^54^ This organization is viewed as an evolutionary ancestor of the more complex mammalian Golgi‐cisternae.^54^ The large size and centralization of the Golgi in SCs may be an indication of very high membrane traffic turn‐over in these cells.^57^

The presence of Rab6 on non‐Golgi compartments has also been reported for other secretory cells, like the cells of the SGs.^37^, ^38^ Interestingly, in SGs, the Rab6 non‐Golgi compartments are localized close to the apical membrane and are thought to be involved in the apical secretion of saliva constituents.^37^, ^38^ We tested the possibility that Rab6‐VLCs are traffic checkpoints for secreted proteins by expressing a reporter protein, Tomato^Myr^, in SCs. Consistent with our hypothesis, Tomato^Myr^ co‐localizes with Rab6‐VLCs, indicating that Tomato^Myr^ is transported via these compartments. However, unlike in the SGs, Rab6‐VLCs are not observed in close proximity to the apical plasma membrane; thus, it seems unlikely that they are a final secretory compartment before apical secretion. More probably, we believe Rab6‐VLCs may represent an early compartment on the route towards secretion. This is supported by the fact that Rab6 VLCs are formed before all other VLCs and knockdown of Rab6 results in the loss of all other VLCs in the SCs.

Interestingly, we found that some Rab6‐VLCs are marked by Rab11 domains.^38^, ^39^ Rab11 is another core‐Rab protein^35^, ^36^ and has been shown to regulate multiple membrane recycling routes.^63^, ^64^ Examining the Tomato^Myr^ marker in lines expressing differentially tagged Rab6 and 11, we were able to show the marker in both Rab6‐ and Rab6/11‐VLCs. This is consistent with previous studies, where it was shown that ectopically expressed Rab11 marks a subset of densely filled vacuoles in SCs that contain secreted molecules like ANCE^30^, ^65^ and DPP.^30^ Combining these results with our finding that *rab6* knock‐down in SCs prevents the formation of Rab11‐VLCs, we conclude that some Rab11‐VLCs are probably downstream compartments involved in the same secretory pathway as Rab6. Furthermore, we found additional small Rab11‐positive (but Rab6‐negative) punctae in close proximity to the apical membrane, suggesting other Rab11‐dependent roles in apical membrane recycling.^37^, ^38^, ^63^, ^82^

Rab19 is another Rab protein that localizes close to the apical membrane in the *Drosophila* SG.^37^ in vitro interaction experiments have shown that Rab19 can interact with the apical adhesion molecule, Pollux, leading some to suggest a role for Rab19‐vesicles in apically directed secretion.^37^, ^83^, ^84^ Here, we show that Rab19 is strongly expressed in the AG and is associated with apically localized VLCs containing our secretion markers (Tomato^Myr^, CG1656 and CG17575). Rab19 is also localized to a small portion of the Golgi apparatus. Because of these findings, it is tempting to propose a role for Rab19‐VLCs in apical secretion in the SCs. Yet this interpretation is confounded by the fact that Rab19 knockdown shows no effect on the PMR, even though no VLCs remain that are marked by Rab19. There are a number of possible explanations for this discrepancy. First, it is possible that Rab19 does not play a role in apical secretion. Evidence presented here suggests that Rab19‐VLCs may differentiate directly from Rab7 compartments^66^, ^67^ as knockdown of Rab7 prevents the formation of Rab19‐VLCs. Therefore, Rab19‐VLCs might not be secretory, but rather endocytic, lysosomal or recycling in nature. Molecules like CG1656 and CG17575, which are known to be part of the seminal fluid^16^, ^26^, ^85^, ^86^ might be transported to the lumen without Rab19‐VLCs, and any of these molecules found in Rab19 compartments could be the result of endocytosis or being targeted for lysosomal destruction. It is even possible that, given the presence of Rab7/19‐positive VLCs and Rab11/19‐positive VLCs that it may be that Rab19 is part of an endocytic route on the way to recycling.^87^, ^88^

Alternatively, Rab19‐VLCs could be secretory and simply are not required for the PMR. Previously, we have shown that the VLCs are not required to secrete some SFPs (including CG1656 and CG17575) to the lumen, but that these proteins are incorrectly modified in mutant backgrounds lacking the VLCs.^26^, ^31^, ^71^ Because loss of these proteins causes the same PMR phenotype as the lack of VLCs, it has been suggested that the modification of these proteins or other proteins with which these proteins interact is the cause of the PMR phenotype. Thus, even if VLCs normally play a role in secretion, there are redundant pathways that can transport these proteins to the lumen, though incorrectly modified. Given this, it is possible that Rab19‐VLCs are organelles on the route towards apical secretion but are not critical (or are redundant) in the modification of the proteins required for the PMR phenotype.

Unlike the knockdown of Rab19, the absence of Rab7 results in the loss of the female long‐term PMR.^16^, ^26^, ^85^, ^86^ As Rab7 is required for proper Rab19‐VLC formation, we originally thought that they would be part of the same pathway, and thus, share the same phenotype. This does not seem to be the case. To explain the discrepancy between the relationships we find between Rab7 and Rab19, and the differences in PMR phenotypes, we believe that the knock down of *rab7* might simply be blocking all endocytic traffic, and that this blockage could lead to the loss of the long‐term PMR through indirect mechanisms. Indeed, Rab7 depletion has been shown to ultimately lead to cell lethality in many other systems.^88^, ^89^, ^90^, ^91^

We have shown that, in male SCs, there are a number of different large, intracellular compartments that can be distinguished by their Rab association. Rab6 seems to be required to establish and maintain two independent trafficking routes (Rab6 to Rab11 and Rab6 to Rab 7/19). Both transport pathways intersect each other at the Golgi apparatus, but only one branch, where Rab6 cooperates with Rab11, might be essential for the transport of SFPs involved in a long‐term PMR.^29^, ^30^ In addition to the work presented here, we have examined the entire Rab machinery in the AGs along with a battery of other protein markers that are accessible through our online resource (https://flyrabag.genev.unige.ch). This work should facilitate future studies on the AGs and on protein trafficking, paracellular transport and the development/organization of membrane identities.

## 4 METHODS

### 4.1 Fly stocks

Male collections were performed at 25°C. *D1‐Gal4* was generated in the lab,^26^ YFP‐tagged *rabs* (*Yrabs*),^37^ *UAS‐Tomato‐myristoylation*,^44^ *UAS‐Lifeactin‐Ruby* 44 and *UAS‐Golgi‐RFP* 49 lines were provided by S. Eaton's laboratory. *UAS‐Rab6RNAi* (ID100774)^92^, ^93^ and *UAS‐Rab19RNAi* (ID103653)^92^ are available from Vienna *Drosophila* Resource Center and *UAS‐Rab7RNAi* line was from M. Gonzalez‐Gaitan's laboratory (University of Geneva).^94^, ^95^, ^96^ Flies were raised at 25°C in tubes on standard yeast‐glucose media (8.2% w/w yeast, 8.2% w/w glucose, 1% w/w agar, 1.2% v/w acid mix).

### 4.2 Immunochemistry

Unless otherwise stated, accessory glands from 5 to 6‐day‐old males were dissected in ice‐cold Grace's Insect Medium (BioConcept), fixed for 20 minutes with 4% Formaldehyde (Sigma) at room temperature and stained with one or more of the following antibodies over‐night at 4°C: anti‐Dlg (Developmental Studies Hybridoma Bank [DHSB]), anti‐DE‐cadherin (DHSB), anti‐Rab7 (DHSB) or with Phalloidin‐546 (Life Technologies). Rabbit polyclonal antibodies against CG1656 (1:500) and CG17575 (1:250) were kindly provided by Mariana Wolfner (Cornell University).^86^ All samples were mounted in Vectashield mounting medium with or without DAPI (Vector Labs). The pictures were taken with a Zeiss LSM700 confocal microscope and evaluated using the FIJI^97^ (Laboratory of Optical and Computational Instrumentation [LOCI], University of Wisconsin‐Madison) and IMARIS softwares (Bitplane AG).

### 4.3 Live imaging

Sample were dissected in ice‐cold PBS and mounted in PBS onto a coverslip. Samples were imaged at approximatively 20°C by an OMX V3 BLAZE microscope (GE Healthcare Life Sciences; Figure 2). Deconvolution algorithms were applied to the acquired wide‐field images using the softWoRx 5.5 software package (GE Healthcare Life Sciences).

### 4.4 Determination of the distribution of the Yrab compartments in the secondary cells

The center of mass of the secondary cells was determined by using Fiji software (a secondary cell was surrounded using the “Freehand selection” and the center of mass was determined by “Measurements”) and a circle of 8.90 μm diameter (ie, average diameter of the apical surface of a secondary cells in contact with the lumen) was drawn by using FIJI software drawing tools; this circle corresponds to the “central” location. The “cortical” and “non‐central cytoplasmic” location indicate that compartments are in close proximity to the cellular membrane or not, respectively. The three “Apical” (luminal side), “Medial” and “Basal” (stromal side) portions were determined by counting the number of z‐slices covering the secondary cell height and this number was divided by three.

The expression patterns of the Rab proteins have been described in the secondary cells from three to 7‐day‐old males. Different terms will be used to describe different Yrab‐labeled structures; we use “vacuole‐like compartments” (VLCs) to refer to structures clearly delimited by a fluorescent membrane, whose diameter can vary from 0.3 μm to 8 μm. The term “small compartments” is used for features >0.5 μm, which are homogeneously fluorescent, and “punctate” for distinct structures <0.5 μm in diameter. Finally, “diffuse” is used for spread out signal without visible particulate structures.

### 4.5 Receptivity and egg laying assays

New‐born virgin males from the different genotypes were put in fresh tubes with dry yeast and stored at 25°C for 5‐7 days, 12/12 hours dark/light cycles. The same was done for virgin *Canton‐S* (CS) females. On the day before the assay, fresh tubes containing one virgin female collected 5 days earlier were set up and kept at 25°C. On the day of mating, one male was added per female‐containing vial. For the tubes where mating occurred, the males were removed and the females were kept for receptivity and egg laying assays at 25°C.

Receptivity assay: Mated females were put in fresh tubes and 4 days after mating, one CS male was added into the tube. The tubes where mating occurs were counted, while for the tubes where the flies did not copulate, the males were removed and the tubes were kept for the next receptivity assay, that is, 10 days after the initial mating (6 days later).

Egg laying assay: single females were transferred every day in a fresh tube and the eggs laid were counted (over a period of 10 days).

## Supporting information


**Editorial Process**

**Appendix S1** Supporting Information
**Figure S1** The distribution of TGN markers in the SCs
**Figure S2** The schematic distribution of Rab6‐, Rab11‐ and Rab19‐ compartments in the SCs
**Figure S3** Tomato^Myr^ pulse‐chase experiment in the SCs
**Figure S4** Tomato^Myr^ pulse‐chase experiment in Rab11, Rab7 and Rab19 marked SCs
**Figure S5** SFPs are found in Rab6‐, Rab11‐ and Rab19‐VLCs
**Figure S6** Cd8‐mRFP is transported in Rab6‐ and Rab11‐compartments
**Figure S7** Rab7 and acidic compartments localization in SCs
**Figure S8** Maturation of the Yrab‐labeled VLCs during virgin male adult development
**Figure S9** The depletion of a specific Rab protein can affect the expression level of the other Rabs
**Figure S10** The schematic distribution of Rab6‐, Rab7‐ and Rab11‐compartments in the MCs
**Figure S11** The loss of Rab6 in the SCs affects their cytology and the morphology of the Golgi apparatus
**Figure S12** Crumbs can traffic via VLCs
