# Supplementary material for: Rab‐mediated trafficking in the secondary cells of Drosophila male accessory glands and its role in fecundity
Source: Traffic. 2018 Dec 26;20(2):137–51. doi: 10.1111/tra.12622 (PMC6492190; doi:10.1111/tra.12622)

Rab-mediated trafficking in the secondary cells of *Drosophila* male accessory glands and its role in fecundity

Rab proteins and male fecundity

E. Prince[1, 2+], B. Kroeger[3], D. Gligorov[1, 5+], C. Wilson[3], S. Eaton[2, 4], F. Karch[1*], M. Brankatschk[2+, 4*] and R.K. Maeda[1*]

Supplemental Information

Supplemental Materials and Methods

Fly stocks

Male collections were performed at 25°C. The *UAS-Arf79F-GFP* line[1] was provided by S. Eaton's laboratory; *w; D1-Gal4, Gal80^{TS}* was generated in the lab; *w;UAS-Cherry^{NLS}* [2], *Crb::GFP* [3] and *Dlg::RFP* [100] was shared with M. Brankatschk's group. The line *w; D1-Gal4::UAS-GFP* line has been characterized in [26] and the *UAS-CD8-mRFP* line was shared by E. Knust's group.

Pulse-chase experiments

D1-Gal4, Gal80^{TS} flies were crossed with *UAS-Tomato^{Myr}* and *UAS-Cherry^{NLS}* at 18°C. In the progeny, 0-1 day-old virgin males were collected and kept aging at 18°C. After 6-8 days, some males were transferred to 29°C and dissected 2h, 4h, 8h and 24h after temperature-shift. Accessory glands were dissected, fixed and stained for 2 hours at room-temperature with DAPI (Invitrogen). After rinsing and washing in 0.1% Triton X-100/PBS solution, all the samples were mounted and imaged (see below, Immunochemistry part).

Immunochemistery

Accessory glands from 5-7 days-old males were dissected in ice-cold Grace's Insect Medium (Sigma), fixed for 20 minutes with 4% Formaldehyde (Sigma) at room temperature and stained with one or more of the following antibodies over-night at 4°C: anti-GFP

(Invitrogen), which was beforehand pre-absorbed, DAPI (Invitrogen), anti-CG1656 and anti-CG17575 (kindly provided by M. Wolfner)[4]. All samples were mounted in Vectashield mounting medium (Vector Labs). The pictures were taken with a Zeiss LSM700 inverted confocal microscope and evaluated using the FIJI[5] Laboratory of Optical and Computational Instrumentation (LOCI), University of Wisconsin-Madison, USA).

Lysotracker staining

Imaging was performed as previously reported in[6].

Quantification of YRab signal in the SCs

Endogenous YFP fluorescence levels in the secondary cells were determined using the FIJI software. For each image slice (of 0.8μm), from the apical to the basal side, the cell area was determined and the fluorescence intensity (IntDen) in this area was measured by FIJI. The background was subtracted for each slide in FIJI giving the Corrected Total Cell Fluorescence (CTCF) value for each section. All the negative values were automatically changed to zero using Microsoft Excel software. To obtain the total fluorescence per cell, the CTCF values for each confocal section of a cell were added. Canton S or *UAS-RabRNAi/+* accessory glands were used for background subtraction. Error bars, Standard Error of Mean (SEM). For Fig. S7, an unpaired *t*-test was performed to compare values of *rab* knockdown glands with *wild-type* glands; n_{cells}= 79-460 (*i.e.* 18-70 accessory glands). For each condition, each n_{cell} is indicated in the chart bars.

Determination of the distribution of the YRab compartments in the MCs

A main cell was surrounded by using "Freehand selection". The "cortical" and "cytoplasmic" location indicate that compartments are in close proximity to the cellular membrane or not, respectively. The three "Apical" (luminal side), "Medial" and "Basal" (stromal side) portions were determined by counting the number of z-slices covering the main cell height and this number was divided by three (Figs. S8A-B).

The expression patterns of the Rab proteins have been described in the main cells from three to seven days-old males. Different terms will be used to describe different Rab-marked structures; we use "small compartments" for features >0.5μm, which are homogeneously fluorescent, and "punctate" for distinct structures <0.5μm in diameter. Finally, "diffuse" is used for spread out signal without visible particulate structures (Figs. S8C-F).

RAB Finder website

Among the YRab library generated by Dunst et al. (2015)[7], 16 Rabs are expressed in the SCs, 14 are expressed in MCs and 3 are expressed in the synaptic buttons of the muscle cell-layer. The distribution of each of these 33 Rabs has been documented and annotated on the website. Some of the localization summaries are also presented here in Figs. S2, S4 and S7. Each of the *Yrab* accessory glands was probed for YFP (green), stained for Dlg or DCAD (magenta) and DAPI (dark-blue). Rabs are clustered depending on the structures, the location on the X- or Y-axis and on the accessory gland-cell type where the Rab can be found. For the Rabs expressed in MCs and/or SCs, confocal images along the Y-axis and schematic distribution are available on the website. Sagittal SC views and confocal stack projections of *Yrabs* accessory gland epithelium or muscle layer can also be found.

References

1. Shao W, Wu J, Chen J, Lee DM, Tishkina A, Harris TJC. A Modifier Screen for Bazooka/PAR-3 Interacting Genes in the Drosophila Embryo Epithelium. *PLOS ONE*. 2010;5(4):e9938. doi:10.1371/journal.pone.0009938

2. Förster D, Luschnig S. Src42A-dependent polarized cell shape changes mediate epithelial tube elongation in Drosophila. *Nat Cell Biol*. 2012;14:526.

3. Huang J, Zhou W, Dong W, Watson AM, Hong Y. Directed, efficient, and versatile modifications of the Drosophila genome by genomic engineering. *Proc Natl Acad Sci*. 2009;106(20):8284. doi:10.1073/pnas.0900641106

4. Ravi Ram K, Wolfner MF. A network of interactions among seminal proteins underlies the long-term postmating response in Drosophila. *Proc Natl Acad Sci U S A*. 2009;106(36):15384-15389. doi:10.1073/pnas.0902923106

5. Schindelin J, Arganda-Carreras I, Frise E, et al. Fiji - an Open Source platform for biological image analysis. *Nat Methods*. 2012;9(7):10.1038/nmeth.2019. doi:10.1038/nmeth.2019

6. Redhai S, Hellberg JEEU, Wainwright M, et al. Regulation of Dense-Core Granule Replenishment by Autocrine BMP Signalling in Drosophila Secondary Cells. Taghert PH, ed. *PLoS Genet*. 2016;12(10):e1006366. doi:10.1371/journal.pgen.1006366

7. Dunst S, Kazimiers T, von Zadow F, et al. Endogenously Tagged Rab Proteins: A Resource to Study Membrane Trafficking in Drosophila. *Dev Cell*. 2015;33(3):351-365. doi:10.1016/j.devcel.2015.03.022

Figure S1. The distribution of TGN markers in the SCs

A confocal reconstruction of a sagittal view of a representative SC (surrounded by a white dashed line) with the apical-basal (Ap-Ba) axis shown as double-ended arrows. Scale bars, 5µm. White asterisks mark labeled vacuoles.

(A) SCs expressing Golgi-RFP were probed for RFP (red) and Dlg (magenta).

(B) *Yrab6* SCs were probed for YFP (yellow) and DAPI (dark blue).

(C) SCs expressing Arf79F-GFP were probed for GFP (green), Dlg (magenta) and DAPI (dark blue).

Figure S2. The schematic distribution of Rab6-, Rab11- and Rab19- compartments in the SCs

Shown are schematic localization of Rab6 (A, n_{cell}=19), Rab19 (B, n_{cell}=13) and Rab11 (C, n_{cell}=5). Key to the symbols used in all schematic representations of Rab localization is present in D. The percentage indications for each structure are defined as follows: for the VLCs, each circle indicates that approximately 10% of the RabX-VLCs in a SC are in this indicated zone. For the small compartments and the punctate, the percentage indicates the proportion of the structures in the indicated zone on the total number of YrabX-labelled compartments (*i.e.* VLCs + punctate + small compartments) per cell. For the diffuse and central mass, the color gradients indicate the percentage of cells with this structure in that zone.

Figure S3. Tomato^{Myr} pulse-chase experiment in the SCs

SCs expressing Tomato^{Myr} (Aa-Ae) and Cherry^{NLS} (Ba-Be), in a thermo-sensitive-manner via *D1-Gal4, Gal80^{TS}* driver (surrounded by white dashed lines), were probed for Tomato (A; magenta) and Cherry (B; magenta). Shown are confocal sections of 6-8 days-old virgin male accessory glands reared at 18°C (a) and then transferred at 29°C (b-e); they were dissected at different time-points after temperature-shift (b-e). Tomato^{Myr}-VLCs are shown by red asterisk (Ad-Ae), scale bars = 7µm.

Figure S4. Tomato^{Myr} pulse-chase experiment in Rab11, Rab7 and Rab19 marked SCs

SCs expressing Tomato^{Myr} under *D1-Gal4, Gal80^{TS}* driver combination and, *Yrab7* (C-C''), or *Yrab19* (D-D'') SCs were probed for Tomato (magenta), a Rab11 antibody *rab11* (green; A-A"; B-B'') and YFP (green; C-D") at different time points after induction (8 hours post induction (A-A", 24 hours post induction (B-D"). Shown are confocal sections of 6-

8 days-old virgin male accessory glands reared at 18°C and then transferred at 29°C for induction. Tomato^{Myr}-VLCs are shown by red asterisks, Rab11-VLCs are shown by blue asterisks, Rab7-VLCs are shown by orange asterisks and Rab19-VLCs are shown by grey asterisks. VLCs that show colocalization between Rabs and Tomato^{myr} are shown by white asterisks. Scale bars = 7μm.

Figure S5. SFPs are found in Rab6-, Rab11- and Rab19-VLCs

Yrab6 (A-A''; D-D''), *Yrab11* (B-B''; E-E'') and *Yrab19* (C-C''; F-F'') SCs were probed for YFP (green) and stained with anti-CG1656 (magenta; A-C'') or anti-CG17575 (magenta; D-F''). Shown are confocal sections of accessory glands (0.8μm; *Yrab6*, apical; *Yrab11*, basal; *Yrab19*, apical), SC shape is outlined (white dashed line) and asterisks label VLCs (pink, Yrab6; blue, Yrab11; grey, Yrab19). Red lozenges show CG1656 and CG17575 structures found in VLCs or surrounding VLCs; scale bars = 7μm.

Figure S6. Cd8-mRFP is transported in Rab6- and Rab11-compartments

(A-D'') Shown are confocal intersections of single secondary cells (0.8μm; A-A'', Basal; B-B'', C-C'' and D-D'', Medial) from *D1>>mCD8-RFP; Yrab6* (A-A''), *D1>>mCD8-RFP; Yrab7* (B-B''), *D1>>mCD8-RFP; Yrab11* (C-C'') or *D1>>mCD8-RFP; Yrab19* (D-D'') flies. AGs were probed for YFP (green; A- D), for RFP (magenta; A'- D') and for DAPI (dark blue). Asterisks indicate VLCs (pink, Yrab6; orange, Yrab7; light blue, Yrab11; grey, Yrab19; red, RFP; white on the merged pictures for VLCs that co-express YFP and RFP). The arrowhead (C'') indicates an intra-VLC compartment co-expressing RFP and YFP. Scale bars = 7μm.

Figure S7. Rab7 and acidic compartments localization in SCs

(A, B) *Yrab7* (A) and *Yrab19* (B) SCs were probed for YFP (green) and stained with Lysotracker (magenta). Rab7-VLCs are typically stained by LysoTracker Red. Shown are confocal stack sections of live accessory glands. SC shape is outlined (white dashed line), asterisks label VLCs (orange, Yrab7; grey, Yrab19; red, Lysotracker; white, for colocalization), scale bars = 5μm.

(C) Schematic Rab7 localization in SCs (n_{cell}=8). Abbreviations: Cort, cortical; NC-Cyto, non-central cytoplasmic; Cent, central (for legend, see the right box). A key to the symbols used in the schematic representation of Rab7 localization is present next to this plot. The percentage indications for each structure are defined as follows: for the VLCs, each circle

indicates that approximately 10% of the Rab7-VLCs in a SC are in this indicated zone. For the punctate, the percentages indicate the proportion of these structures in the indicated zone on the total number of Rab7-labelled compartments (*i.e.* VLCs + punctate + small compartments) per cell. For the diffuse features, the color gradients indicate the percentage of cells with this structure in that zone.

Figure S8. Maturation of the Yrab-labelled VLCs during virgin male adult development.

Yrab6 (A), *Yrab7* (B), *Yrab11* (C) and *Yrab19* (D) secondary cells (traced by white dashed lines) were probed for YFP (green), stained for Dlg (magenta) and DAPI (dark-blue). Shown are confocal sections of virgin male accessory glands at different post-eclosion time-points (a-d). VLCs are shown by asterisks (pink, Yrab6; orange, Yrab7; light blue, Yrab11; grey, Yrab19). Scale bars, 5μm.

Figure S9. The depletion of a specific Rab protein can affect the expression level of the other Rabs.

Histograms representing the expression of Yrab6, Yrab7, Yrab11 and Yrab19 in secondary cells probed using YFP fluorescence. Shown are YFP intensities in a *wild-type* condition (black), in the absence of Rab6 (pink), Rab7 (orange) and Rab19 (grey); n_{cells} are indicated on each column. Statistics, $p<0.0001$ (****); p= 0.0263 (*); $p>0.5$ (non-significant, ns); unpaired *t*-test.

Figure S10. The schematic distribution of Rab6-, Rab7- and Rab11- compartments in the MCs

(A, B) Schematic depiction of main cells (magenta) flanking a secondary cell (cyan). We divided MCs into three zones on the y-axis: Apical, Medial and Basal (A, sagittal view) and in two areas on the x-axis: Cortical (Cort., green) and Cytoplasmic (Cyt., blue) (B; top view).

(C-F) Shown are schematic localization of Rab6 (D, n_{cell}=13), Rab7 (E, n_{cell}=7) and Rab11 (F, n_{cell}=3). Rab19 is not represented since it cannot be detected in the main cells. Key to the symbols used in all schematic representations of Rab localization is present in C. The percentage indications for each structure are defined as follows: for the small compartments and the punctate, the percentage indicates the proportion of the particular structures in the indicated zone on the total number of YrabX-labelled compartments (*i.e.* punctate + small

compartments) per cell. For the diffuse features, the color gradient indicates the percentage of cells with this structure in that zone.

Figure S11. The loss of Rab6 in the SCs affects their cytology and the morphology of the Golgi apparatus.

Fluorescent microscope image of secondary cells from accessory glands expressing a nuclear GFP (A-A´, green) or a Golgi-RFP marker (B-B´, magenta) in a secondary-cell-specific manner by using the D1-Gal4 driver. GFP and RFP signal were observed in *wild-type* conditions (A, B) and in SCs where Rab6 is depleted (A´, B´). Scale bars, 10µm.

Figure S12. Crumbs can traffic via VLCs

Shown are confocal slices of individual *DLG-RFP;Crb-GFP* (A-A'', medial), *DLG-RFP* (B-B'', medial), *D1>>Lifeactin-Ruby; Crb-GFP* (C-C'', medial) and *D1>>Tomato*^{Myr}*; Crb-GFP* (D-D'', medial) SCs. AGs were probed for anti-GFP (green), RFP/Ruby (magenta) and DAPI (dark-blue). SC shape is outlined (white dashed line) and yellow asterisks show GFP-positive VLCs; scale bars = 7µm. (D-D'') VLCs labelled by Tomato^{Myr} or/and GFP are depicted by red/white asterisks, respectively.

Fig. S1: The distribution of TGN markers in the SCs

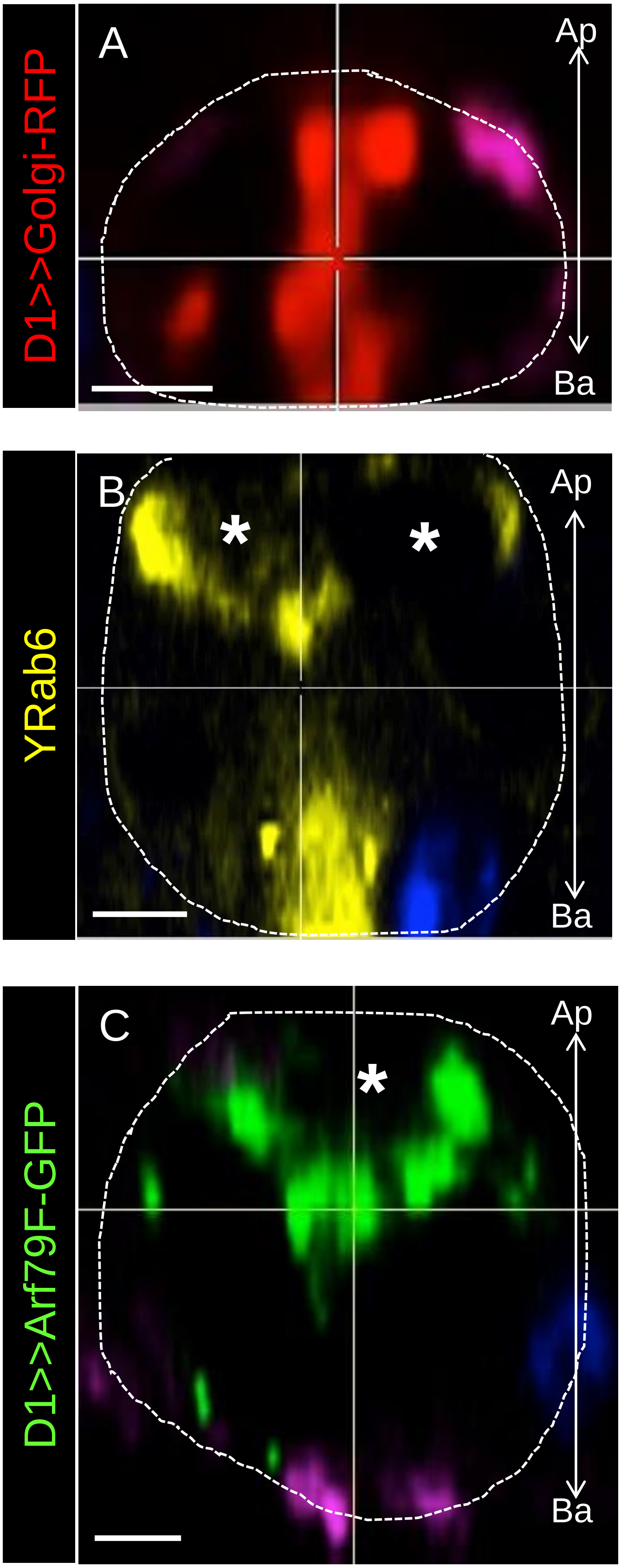

Fig. S2: Schematic representation of Rab6, Rab19 and Rab11 in SCs

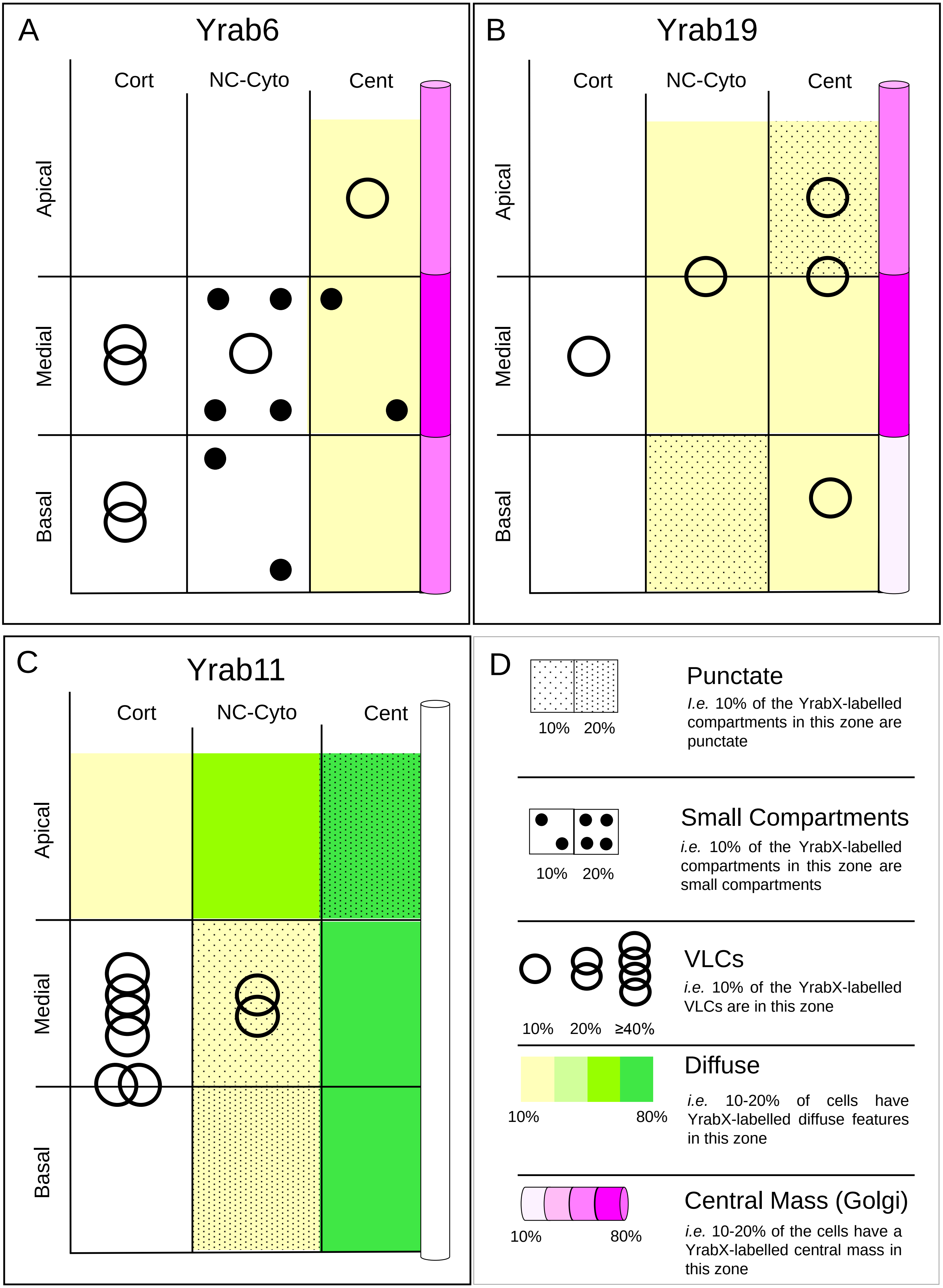

Fig. S3: Tomato^{Myr} pulse-chase experiments in the SCs

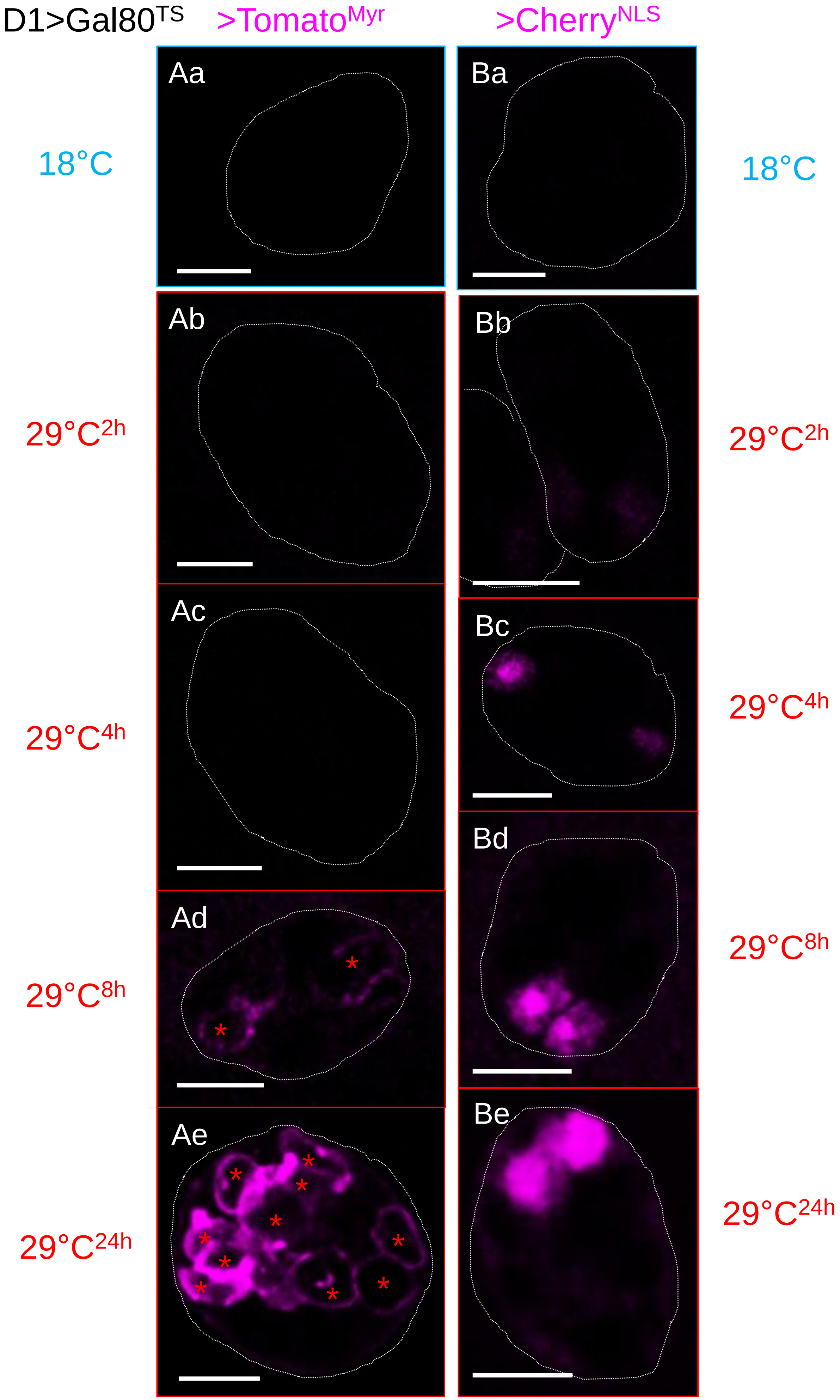

Fig. S4: Tomato^{Myr} pulse-chase experiments in Yrab11, Yrab7 and Yrab19 SCs

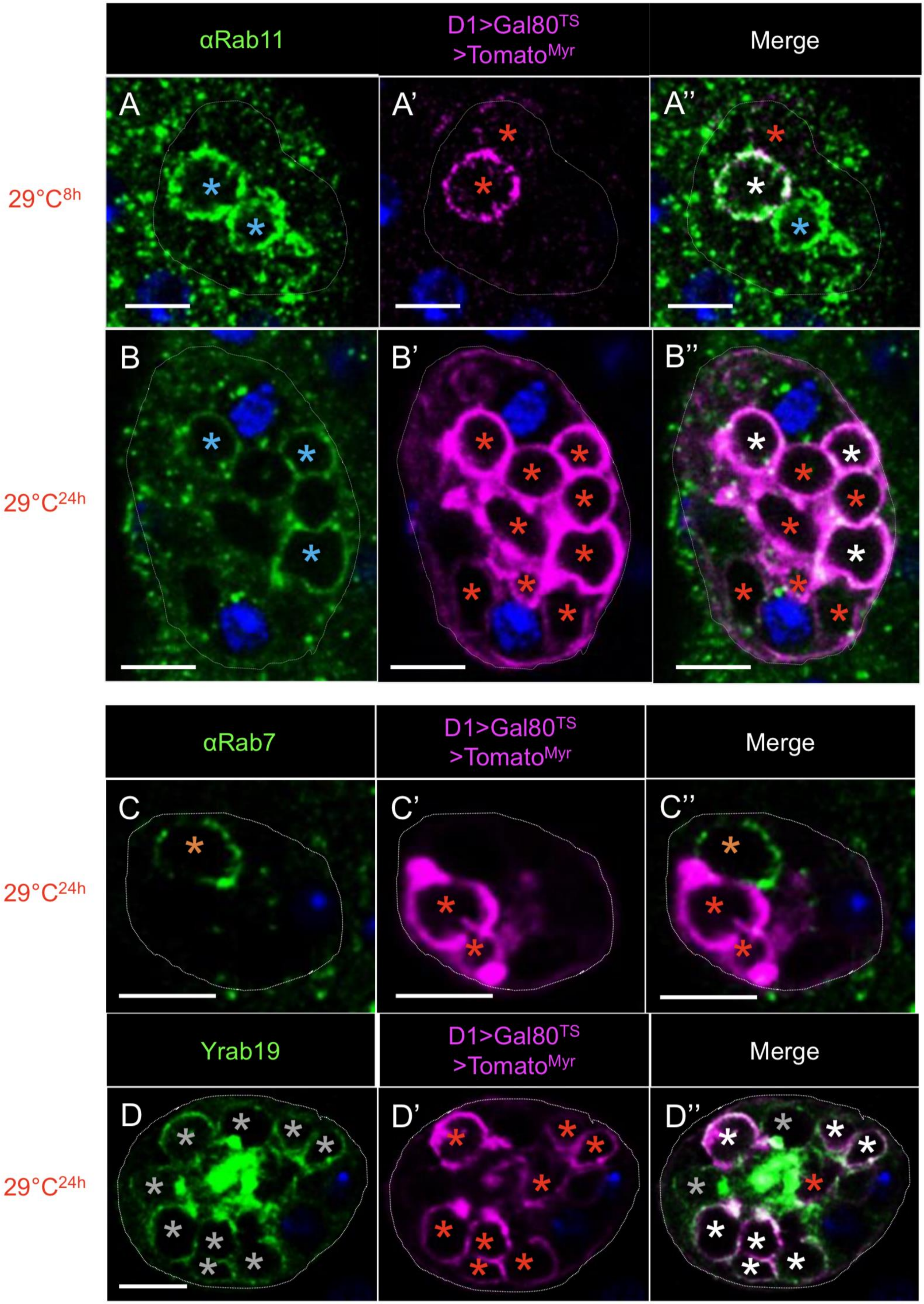

Fig. S5: SFPs are founds in the Rab6-, Rab11- and Rab19-VLCs

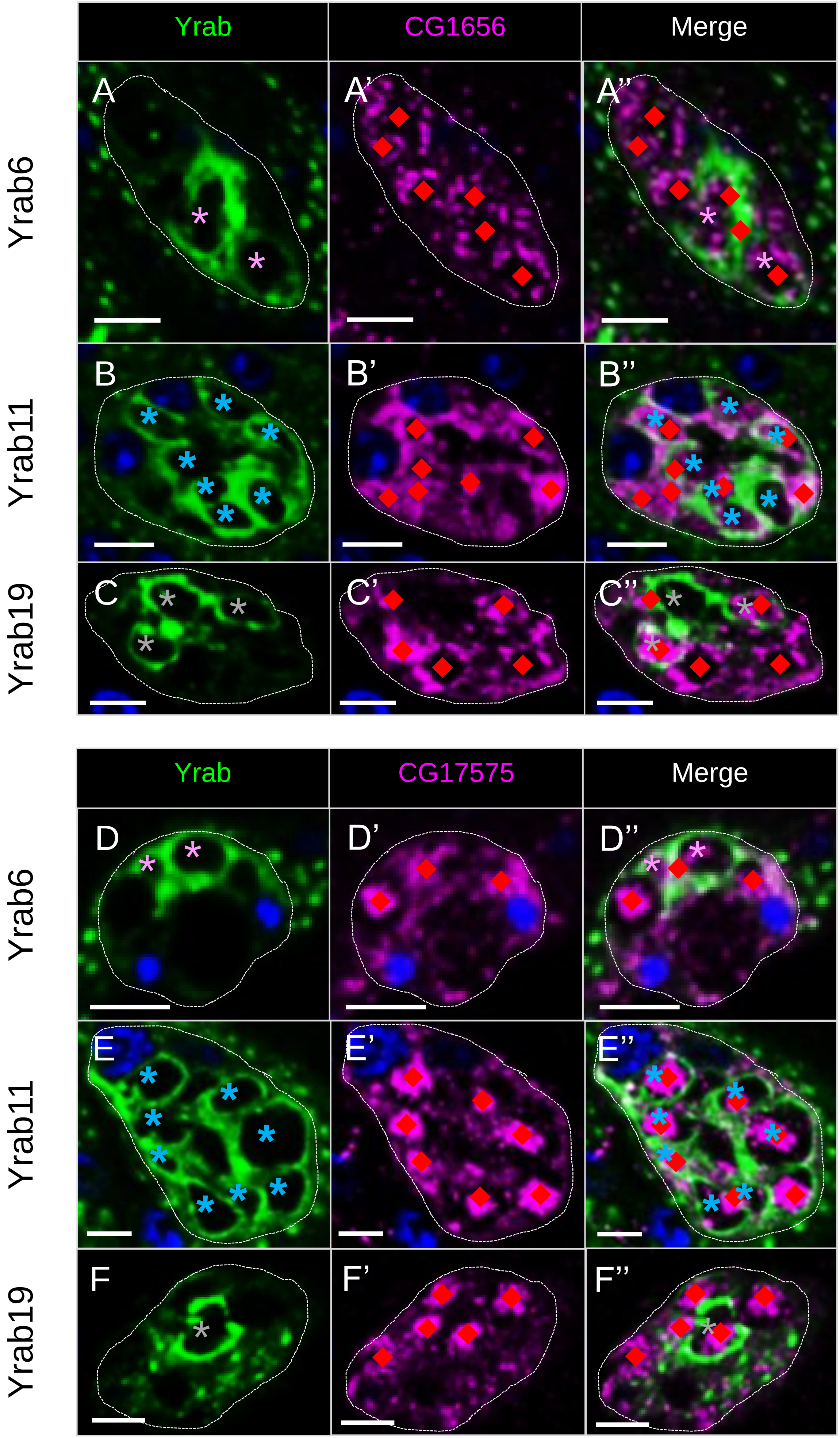

Fig S6. RFP^{TM} is transported in Rab6- and Rab11-compartments

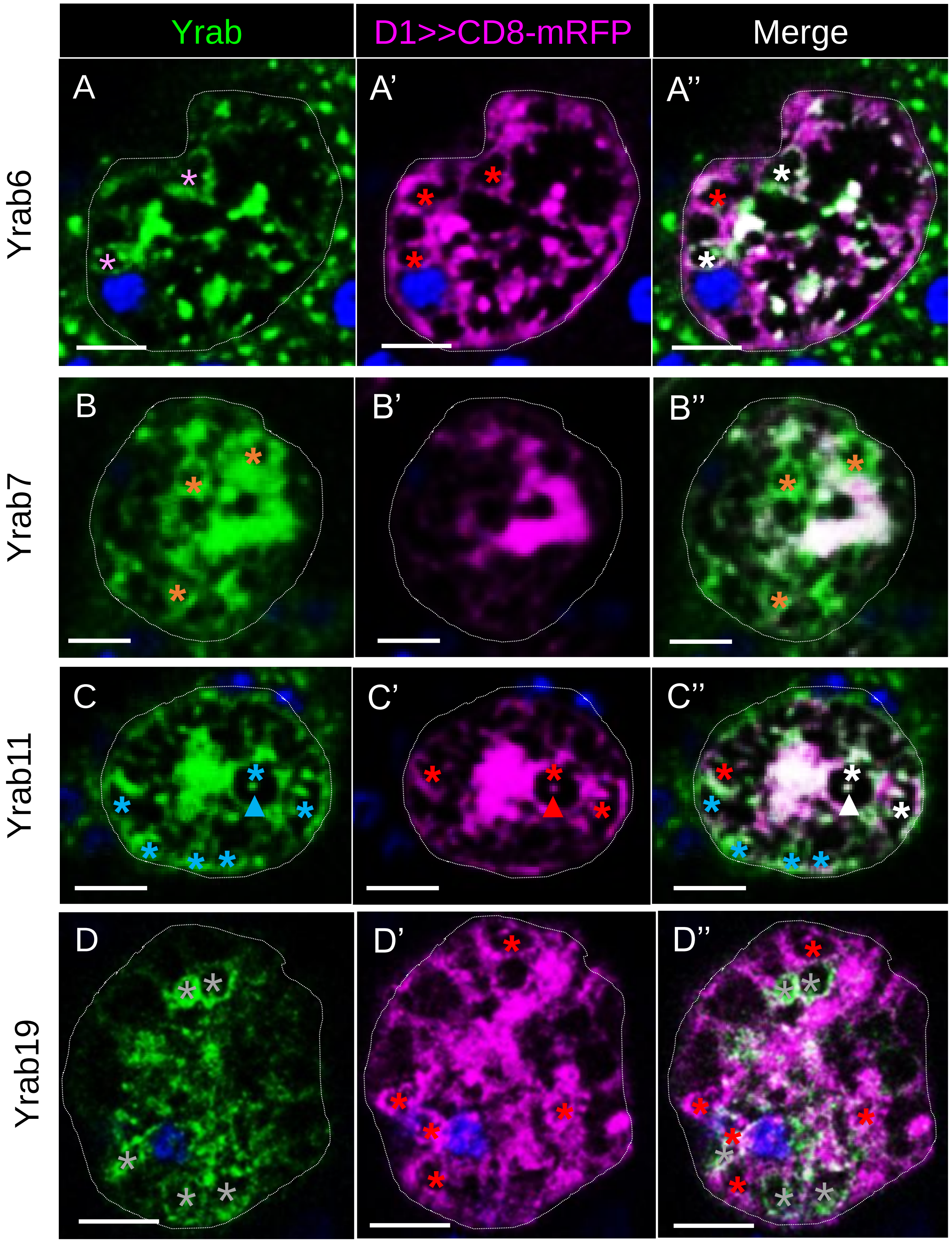

Fig S7. Rab7 and acidic compartments localization in SCs

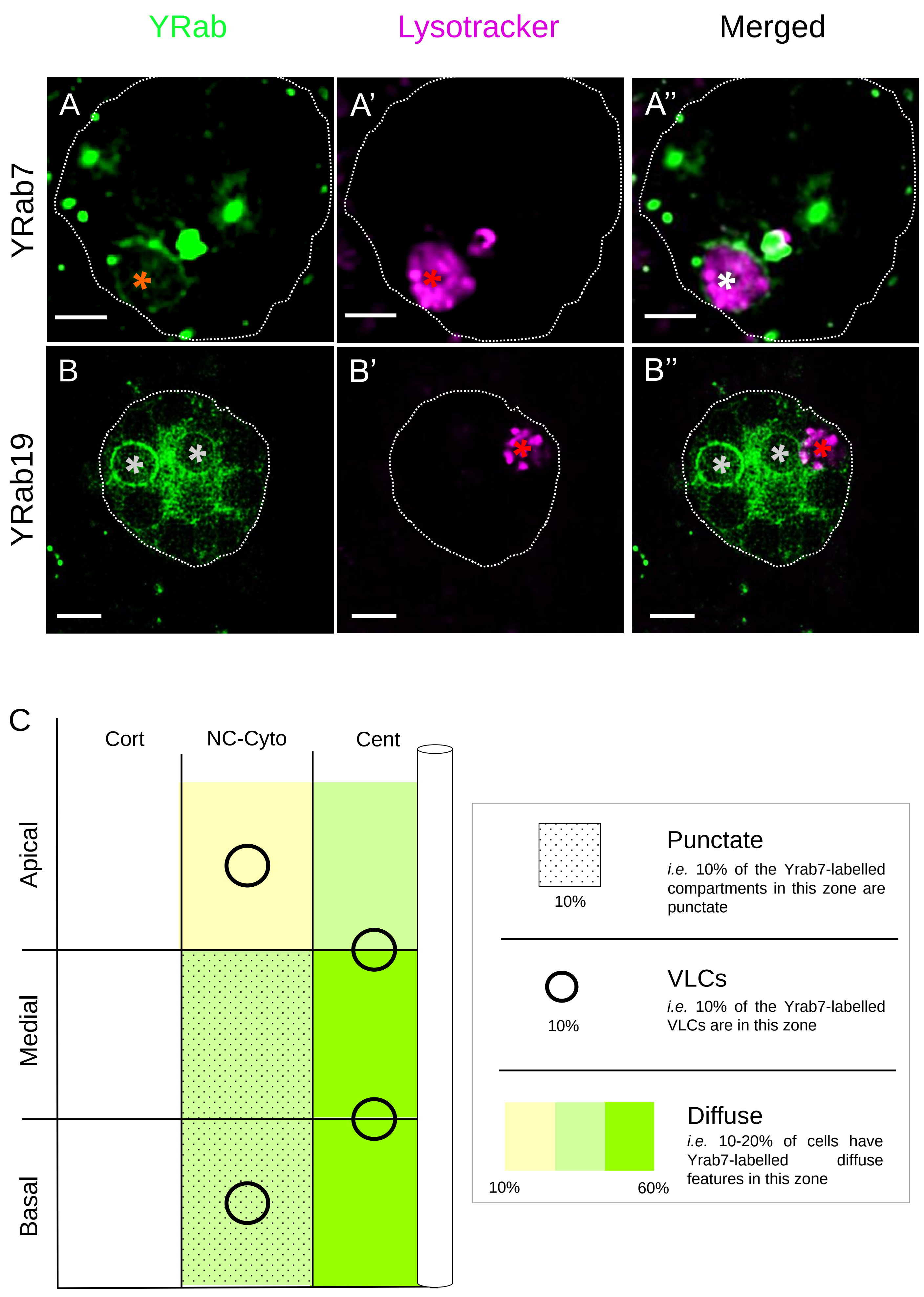

Fig. S8: Maturation of the Yrab-labelled VLCs over virgin male development.

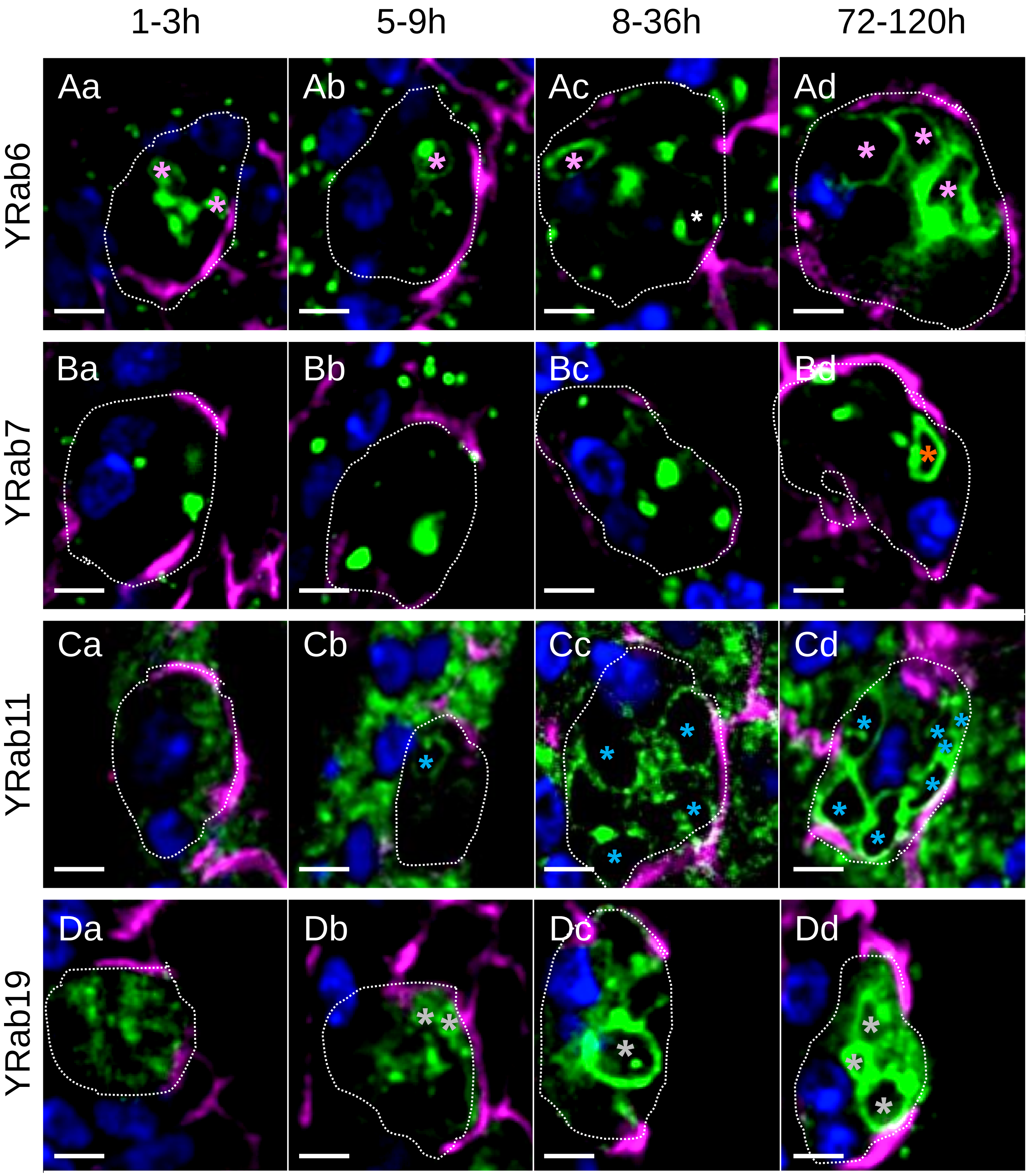

Fig. S9: The depletion of a specific Rab protein can affect the expression level of the other Rabs.

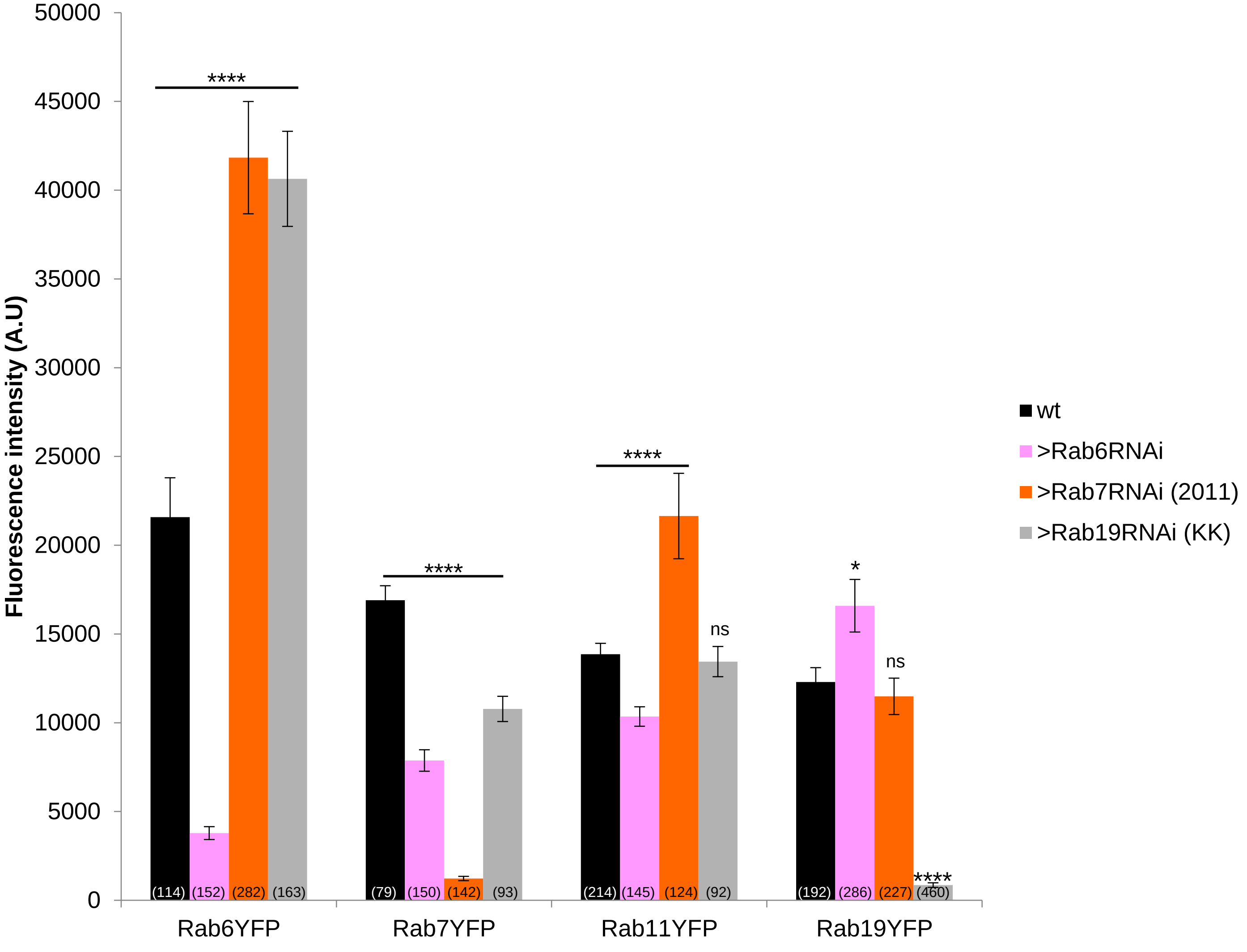

Fig. S10: Schematic representation of the distribution of Rab6, Rab7, Rab11 in the MCs

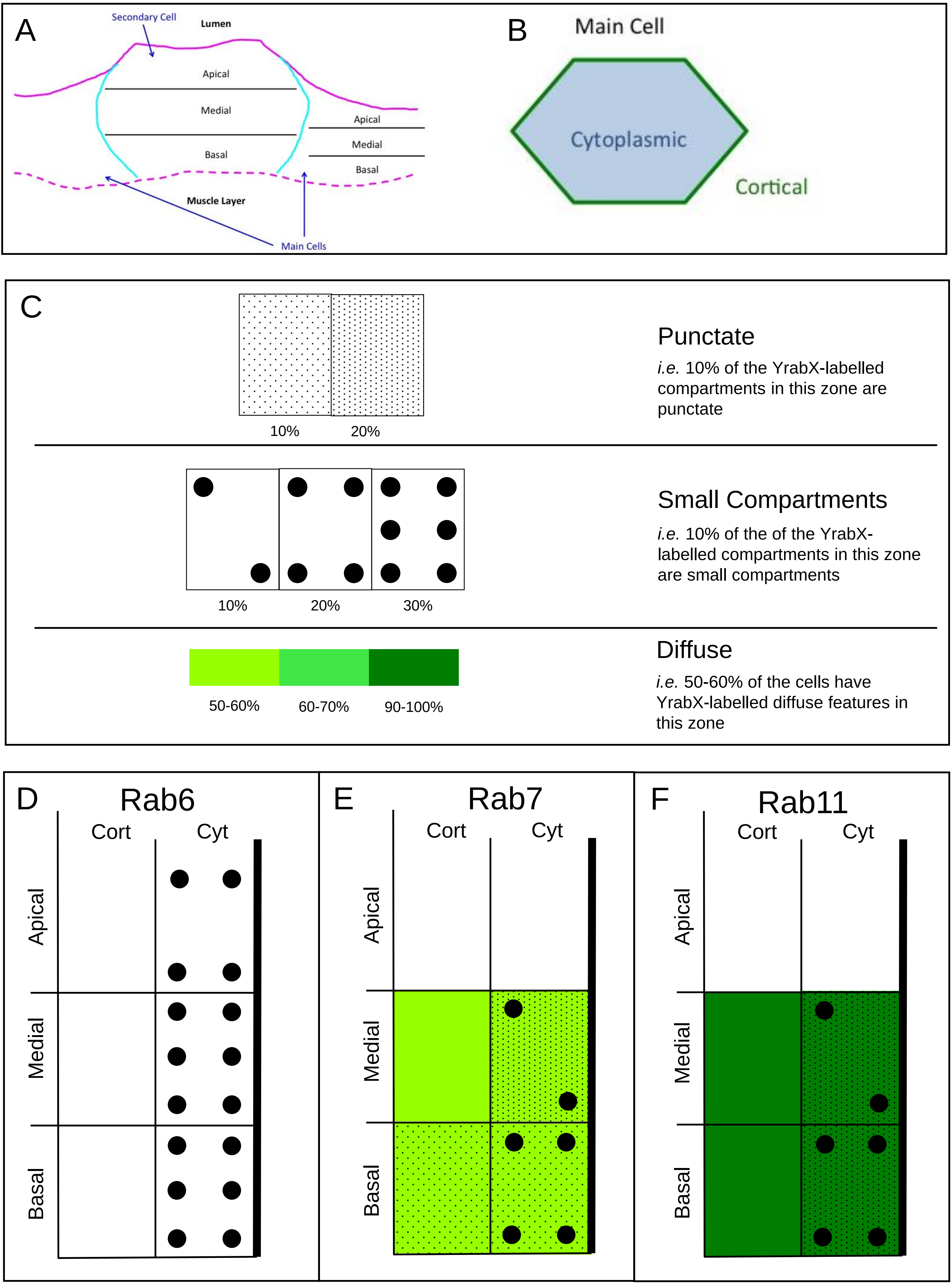

Fig. S11: The loss of Rab6 in the SCs affects their cytology and the morphology of Golgi apparatus.

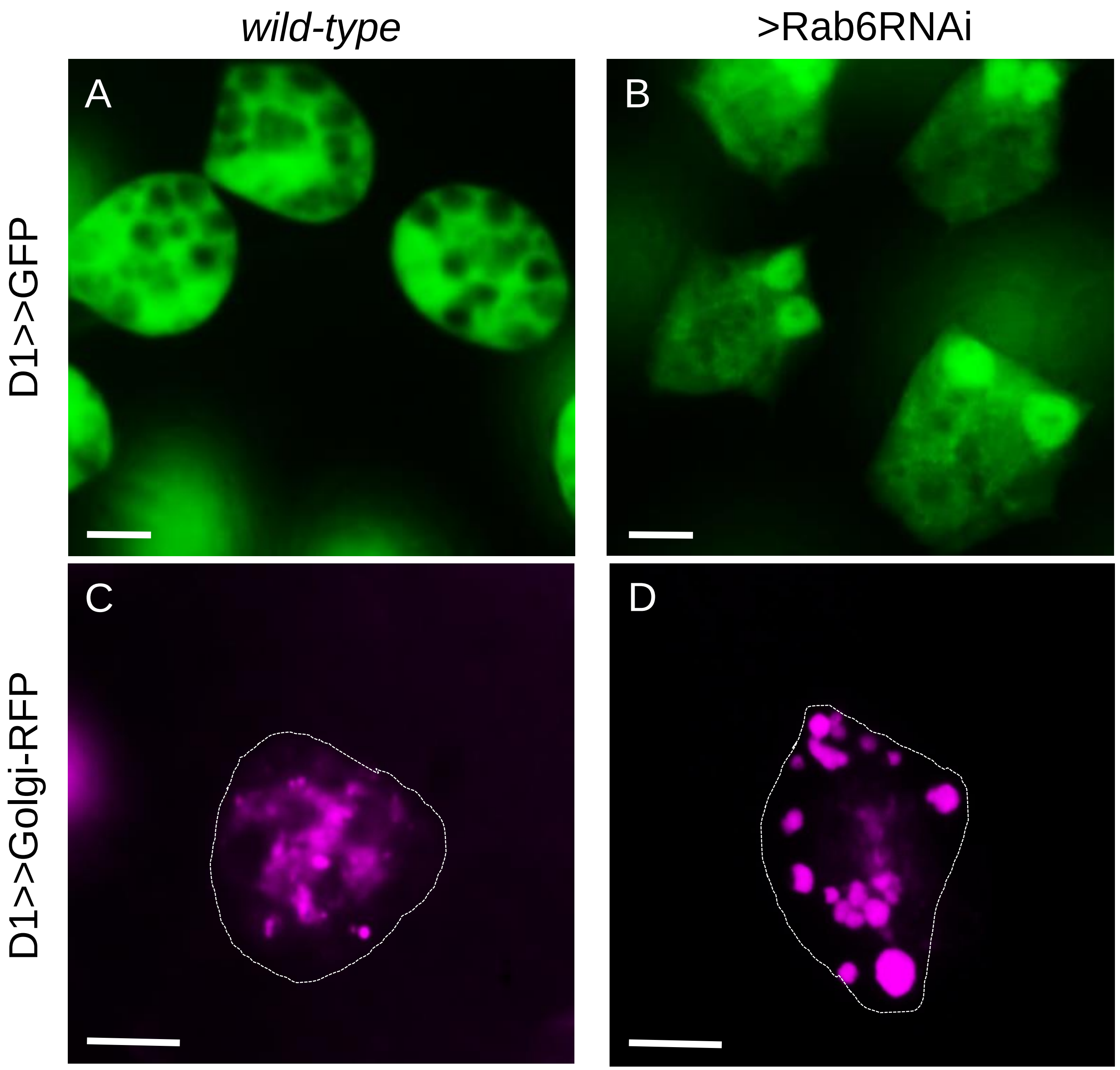

Fig. S12: Crumbs can traffic via VLCs

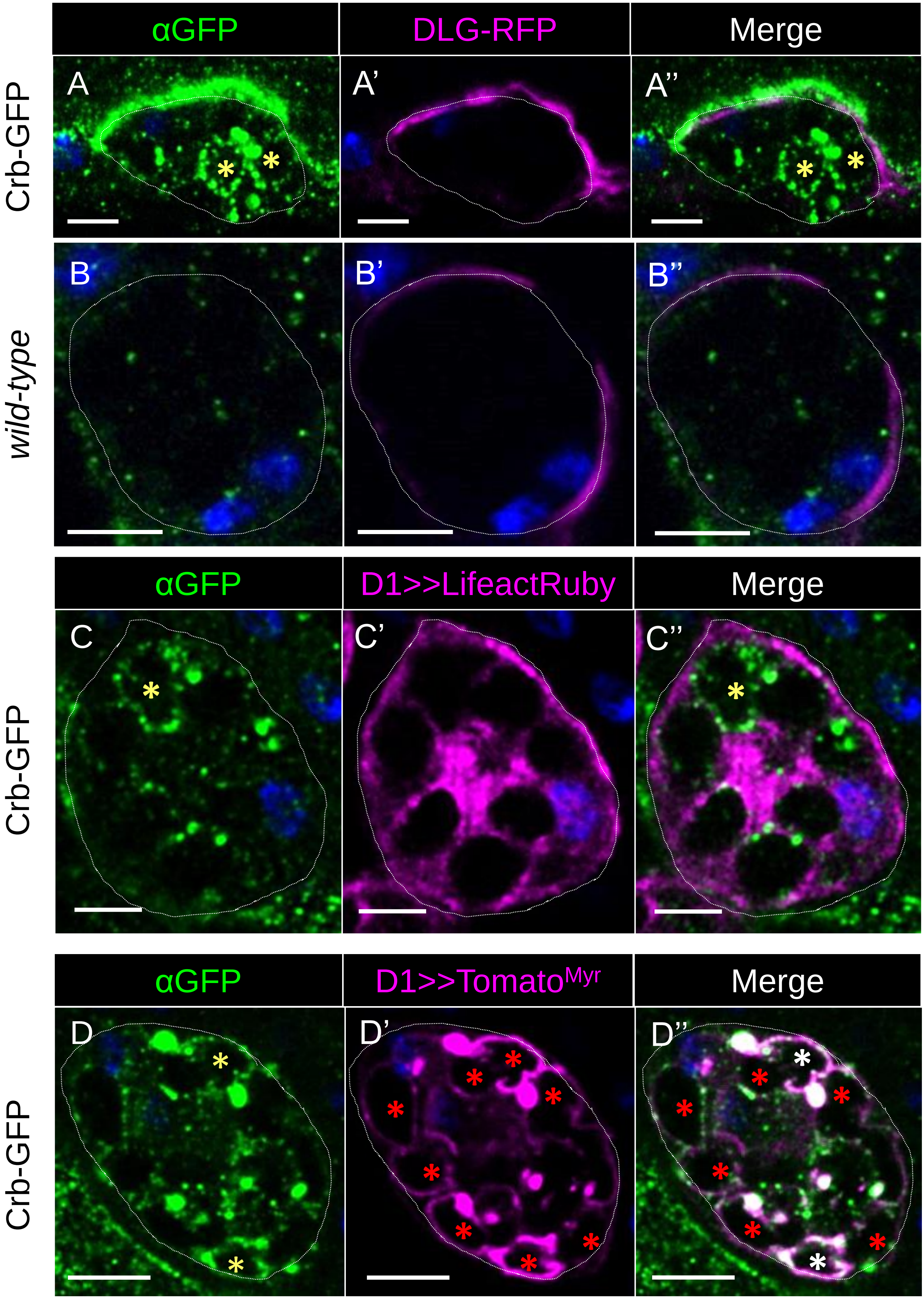

Supplement: Supplementary file 2 — Appendix S1 Supporting Information Figure S1 The distribution of TGN markers in the SCs Figure S2 The schematic distribution of Rab6‐, Rab11‐ and Rab19‐ compartments in the SCs Figure S3 TomatoMyr pulse‐chase experiment in the SCs Figure S4 TomatoMyr pulse‐chase experiment in Rab11, Rab7 and Rab19 marked SCs Figure S5 SFPs are found in Rab6‐, Rab11‐ and Rab19‐VLCs Figure S6 Cd8‐mRFP is transported in Rab6‐ and Rab11‐compartments Figure S7 Rab7 and acidic compartments localization in SCs Figure S8 Maturation of the Yrab‐labeled VLCs during virgin male adult development Figure S9 The depletion of a specific Rab protein can affect the expression level of the other Rabs Figure S10 The schematic distribution of Rab6‐, Rab7‐ and Rab11‐compartments in the MCs Figure S11 The loss of Rab6 in the SCs affects their cytology and the morphology of the Golgi apparatus Figure S12 Crumbs can traffic via VLCs [file TRA-20-137-s001.pdf]
