# Supplementary material for: Rab‐mediated trafficking in the secondary cells of Drosophila male accessory glands and its role in fecundity
Source: Traffic. 2018 Dec 26;20(2):137–51. doi: 10.1111/tra.12622 (PMC6492190; doi:10.1111/tra.12622)
Supplement: Supplementary file 1 — Editorial Process [file TRA-20-137-s002.pdf]

Title:	**Rab-mediated trafficking in the secondary cells of *Drosophila* male accessory glands and its role in fecundity**
Authors:	Elodie Prince, Benjamin Kroeger, Dragan Gligorov, Clive Wilson, Suzanne Eaton, François Karch, Marko Brankatschk and Robert K. Maeda
Article Type:	Original Research Article

Monitoring Editor	Michael S. Marks
Date Submitted	29 March 2018
Date for Decision 1	30 April 2018
Date Resubmitted	27 September 2018
Date for Decision 2	24 October 2018
Accepted	6 November 2018

Decision and Reviews

Dear Dr. Maeda

Thank you for submitting your manuscript "Rab-mediated trafficking in the secondary cells of Drosophila accessory glands" to be considered for publication in Traffic. I asked two colleagues who are experts in the field to review the paper and their verbatim comments are appended below. I agree with the referees that the work presented in this paper will be of significant interest to the readers of Traffic, and that the work is generally very well done. However, both referees raise a few concerns that you will need to address before this paper can be accepted. Although addressing these concerns will require the inclusion of additional data as well as revisions to the text, I believe that you will find the necessary experiments to be straightforward and I look forward to receiving your revised manuscript in the near future. To expedite handling when you resubmit please be sure to include a response detailing how you have addressed each of the referees' concerns.

Sincerely,

Michael S. Marks, PhD
Co-Editor

Referee's Comments to the Authors

Referee: 1

Comments to the Author
In this manuscript, the authors have localized several RAB GTPases (RAB6, RAB7, RAB11 and RAB19) to large vacuole-like structures (VLC) in the secondary cells of Drosophila accessory cells. They also provide evidence that RAB6 controls the formation of VLCs and that its depletion impairs the post-mating response (PMR).

The experiments are carefully done and the data are overall convincing. This study provides interesting information on the biogenesis of VLCs. The authors have also created an online, open-access imaging resource that illustrates the localization of RAB GTPases in the secondary cells. This resource should be of interest and useful for the trafficking community.

Specific comments :

1) A better characterization of the cargoes associated with VLCs positive for a given RAB should have been performed. In the case of RAB6-positive VLC, what is the evidence that Tomatomyr is a secretory marker? Could its secretion be directly monitored? As the secondary cells are polarized, co-localization of RAB6-positive VLCs with apical and basolateral cargoes should precise the role of RAB6. In the case of RAB7/RAB19-positive VLC, whether they are accessible to endocytic markers should be tested.
2) I had an hard time understanding the legends of Figs 2D, 3C, 4C, and 5C. For instance, in Fig. 2D, if I understood correctly, the blue circles correspond to an average of 10% of the RAB11-positive VLCs present in a given zone. There is a total of 8 blue circles. Should not it be 10 (100%). Also, only symbols that are used in the corresponding Fig. should be shown.
3) The authors found surprising that some RAB6-positive VLCs are also labelled with RAB11. However, functional links between RAB6 and RAB11 have been already described (Miserey-Lenkei et al, Traffic 2007, 8 :1385).

Referee: 2

Comments to the Author
The manuscript describes the functional significance of the secondary cells in accessory gland of Drosophila that secretes and contributes factors in the seminal fluid.
My comments are as follows:
1. With reference to the introduction it is adequate, it has all the relevant information as per the demand of the experiments carried out. However, some more knowledge about the different Rab proteins playing specific roles in epithelia other than Accessory Gland epithelium would be useful.
2. The results section is fine, however some important points have not been answered:
From line no.124 onwards experiments regarding analysis of the morphology of cells of AGs have been analyzed using different polarity markers. Here, dE-Cadherin has been used to observe the apical domains of cells, however, Cadherins are basically cell-cell adhesion molecules, so what about the typical markers of the apical domains of the epithelial cells like crumbs or bazooka? Were they also checked? A detailed analysis of these complexes would be helpful to understand the the morphology of the cells better.
3. References are not in the standard format. All the references should follow same pattern. No issue date or month and no issue number is given in standard reference writing.

Author Rebuttal

Dear Dr. Marks,

Thank you very much for handling our manuscript submitted to Traffic entitled, "Rab-mediated trafficking in the secondary cells of Drosophila accessory glands". We were pleased to read the thorough and constructive suggestions given by the reviewers and have attempted to address each of their points. Many of the experiments suggested by the reviewers have led to interesting new findings that have now been included in our revised manuscript in the form of one new figure (Fig. 6) and 6 new supplementary figures (Supp Figs. S3-7 and S12). Other points of clarification have been submitted as an additional file for the reviewers' inspection. Overall, we believe that the additional work and clarifications have addressed the primary concerns of the reviewers and have made a much stronger manuscript.

Thank you again for considering our work for publication in Traffic.

With best wishes,

Robert Maeda

Reviewer 1:
1. A better characterization of the cargoes associated with VLCs positive for a given RAB should have been performed. In the case of RAB6-positive VLC, what is the evidence that TomatoMYR is a secretory marker?

The reviewer is correct. TomatoMyr is not exactly a secreted protein but rather a protein that associates with distinct membranes (including the plasma membrane) (Heinrich et al., 2014; McCabe et al., 1999; Simon and Aderem, 1992). We changed the respective text in the manuscript and it now reads:

"The myristoylated fluorescent protein, TomatoMyr, associates with distinct cellular membranes including the plasma membrane, and its lipid modification prevents free intracellular diffusion 59. Therefore, we presume that TomatoMyr needs to enter the secretory route to reach the cell periphery 44,60,61 (Fig. S3). To test if Rab6-VLCs shuttle proteins along this secretory route, we expressed TomatoMyr in Yrab6 SCs. Our results show that the cortical and NC-cytoplasmic Rab6-positive VLCs are used to transport this reporter protein (Fig. 3D-D''). . . "

Because of the potential limitations of using TomatoMyr as our only secretion marker, we confirmed our results using additional molecules. First, we examined two known seminal fluid proteins (CG1656 and CG17575) that are expressed in the secondary cells and are known to be involved in creating the long-term PMR. These data are presented in supplementary figure 5 and largely confirm the results obtained with TomatoMyr, localizing to Rab6, Rab11 and Rab19 VLCs. These proteins showed particularly strong localization to the Rab11 VLCs, where they often localized to masses in the interior of the VLCs (especially with regards to CG17575). Previous work by one of the groups involved in this manuscript has described these Rab11 VLCs as Dense Core Granules that also contain molecules like ANCE, another cargo molecule. Besides this Rab11 localization, we also found the two ACPs tested (CG1656 and CG17575) in Rab 19 and Rab 6 VLCs. While the staining in Rab19 VLCs looked similar to that seen in Rab11 VLCs (DCG-like), the Rab6 VLC contained more punctate staining. Both ACPs seemed absent from Rab7 VLCs, though Rab7 VLCs were more difficult to visualize in these experiments.

Next, we examined the CD8-RFPTM reporter, a transmembrane protein that should be transported to the plasma membrane. Again, results from the CD8-RFPTM reporter, largely recapitulate our results using TomatoMyr. Interestingly, we found CD8-RFPTM enriched in apical membranes and, during its trafficking, co-localized with Rab6-, Rab11 and potentially some Rab19-VLCs (Fig. S6). It also could sometimes be found WITHIN Rab11-VLCs (Supp Fig. S6C), which may indicate their presence on multi-vesicular bodies (previously described in SCs (Corrigan et al. 2014)). We conclude that CD8-RFPTM is transported in Rab6 and Rab11-positive compartments. The presence of CD8-RFPTM inside a subset of Rab11-VLCs could point to their involvement in recycling and is similar to what we have seen for TomatoMyr (Fig. 4 and Supp Fig. S4). Although we did not detect CD8-RFPTM in Rab7-VLCs, we were able to see co-localization with other non-VLC Rab7-positive compartments (Supp Figs. S6B-B''). We presume that this represents a portion of the reporter that is degraded and therefore, localizes to Rab7-positive late, endosomes. Of note, the massive expression of CD8-RFPTM affects the morphological appearance of SCs - detectable VLCs are smaller and increased in numbers.

Taken together, Rab7-VLCs did not associate with any cargo expected to enter the secretory and/or recycling route. These results are consistent with previous results from the Wilson lab, which showed that Rab7 VLCs did not colocalize with known their cargo markers but are positive for the lysosomal marker, Lysotracker (Supp. Fig. S7A-B).

One difference that we found with these newer experiments is with regards to Rab19 transport of cargo molecules. In our initial experiments with TomatoMyr, we noticed only a very small amount of marker colocalizing with Rab19 VLCs. Here, we have looked at multiple new markers and can confirm that they are present on some Rab19 vacuoles. Repeating our TomatoMyr experiment using the pulse chase conditions discussed below, we do find that TomatoMyr can co-localize with Rab19-VLCs (but not with Rab7-VLCs, Supp Fig. S4C-C'', S4D-D''). Based on these new results, we have modified our interpretation of the role of Rab19 in SCs.

Could its secretion be directly monitored?

To monitor secretion, we performed a series of pulse-chase experiments using TomatoMyr. For these pulse chase experiments, a temperature sensitive Gal80 repressor was introduced into each of the reporter lines. In Drosophila, the temperature-sensitive Gal80TS suppresses Gal4-driven expression at low temperatures. Temperature-shift to 29°C deactivates Gal80TS and allows Gal4-regulated protein production. As each of our markers was expressed under the control of Gal4, the Gal80TS allowed us to precisely control the induction of the fluorescent marker.

To test the quality and specificity of our induction system, we used CherryNLS, expecting that CherryNLS will unlikely appear in VLCs. Without induction only very low levels of CherryNLS are detectable (expression at 17-18˚C). After induction (shift from 18˚C to 29˚C), CherryNLS is enriched in the nucleus and was strongly visible after 4h in SCs confirming the quality of our expression system and protocol (Supp. Fig. S3B).

Examining TomatoMyr after induction showed that the marker localizes to a few VLCs after 8h of induction. By 24

hours of induction, the marker can be seen in numerous VLCs (Fig. S3). We were able to visualize that some of the early VLCs marked by TomatoMyr were Rab11-VLCs (Supp Fig. S4). It is interesting to note that some non-Rab11 vacuoles were also marked by TomatoMyr. These may be Rab6 VLCs, but we were unable to verify this, as recombinant lines containing the Yrab6 marker and TomatoMyr were not obtained. Later, we could find TomatoMyr also labeling Rab19 VLCs (Supp Fig. S4D). Thus, we believe that the transportation of TomatoMyr may be via Rab11 VLCs (and probably Rab6-VLCs), potentially followed by some Rab19-VLCs. For now, we have included much of this data as supplementary figures since we do not have the Rab6 data and do not wish to over-speculate on this issue.

Although we also attempted to perform experiments using Cherrysterol and Cherrysec markers, we unfortunately had limited success, mostly due to the difficulty in generating lines expressing all of the markers in the time frame of this revision; we were unable to generate both the Rab6 and Rab11 lines appropriate for these studies. Still, we present the data we obtained in the additional figures for the reviewers (Reviewer's Fig. R1). Basically, they show that these markers do not localize to Rab7-VLCs, and only have limited overlap with Rab19-VLCs.

As the secondary cells are polarized, co-localization of Rab6-positive VLCs with apical and basolateral cargoes should precise the role of Rab6.

Aside from CG1656 and CG17575 that must be secreted into the central lumen to become part of the seminal fluid, we also tested the apical and basolateral cargoes GFP-tagged Crumbs (Crb, a TYP-I transmembrane receptor required for the establishment and maintenance of apical polarity) and RFP-tagged Disc-large (Dlg, a protein associated with the basolateral membrane). Crb is also present in transport compartments. Unfortunately, technical problems prevented a direct co-localization experiment using our rab alleles since we used the knock-in line crb-gfp (Huang et al., 2009). Therefore, we tested the localization of TomatoMyr and Crb-GFP on VLCs – since TomatoMyr is localized on Rab6-VLCs. Interestingly, Crb-GFP is located on TomatoMyr- VLCs pointing out the association of Rab6-VLCs with apical cargo-transport (Supp Fig. S12D-D''). RFP-Dlg appears to be only associated with the plasma membrane however (Reviewers' Fig. R2)

In the case of RAB7/RAB19-positive VLC, whether they are accessible to endocytic markers should be tested.

As a side note, we would first like to refer you to the newer findings regarding Rab19-VLCs, mentioned above. We originally thought that the connection between Rab7 and Rab19 could mean that Rab19 played a role in endocytosis. However, we find a number of secreted molecules in Rab19 VLCs. This made us reinterpret the role of Rab 19 and Rab7/19 positive VLCs. The Wilson lab previously reported that Rab11 and Rab7 could co-localize on some VLCs (Corrigan et al 2014)). Here, we show that some Rab19-VLCs are also positive for Rab7 (Fig. 6D). Both here and in previous work, Rab7 compartments have been shown to be present in two forms: those stained by Lysotracker and those that do not (Supp Figs. S7A-A"). The Wilson lab has shown (Corrigan et al 2014), that the Lysotracker negative compartments are Rab11/Rab7 positive. Given that all Rab19 compartments are Lysotracker negative, might indicate that these VLCs may be the same compartments also marked with Rab11 and Rab7. Although no true function has been determined for these VLCs, given their association with Rab 11 and 7, they may be recycling in nature. And additionally, we found that expressing CD8-RFPTM in the SCs did not show CD8-RFPTM in Rab7-VLCs (Supp Figs. S5B-B'', see also above). We expected that CD8-RFPTM would be placed into the plasma membrane using the secretory Rab6/11 route and would later get endocytosed with portions of the plasma membrane. Although we do not see CD8-RFPTM associated with Rab7 VLCs, we do find it associated with other Rab7 compartments. This suggests that other Rab7 compartments may be part of the typical endocytic pathway, but that Rab7-VLCs might play a role outside of this pathway, perhaps as a holding compartment. We have discussed some of these points in the discussion of the manuscript.

We did, however, also attempt to answer the reviewer's point directly using A555-Dextran10000 in endocytotic uptake assays. Unfortunately, we could not observe any uptake, probably due to the smooth muscle layer surrounding the basal side of the AG. As removal of these muscle cells was too difficult to perform without damaging the tissue, we had to abandon the further pursuit of these experiments.

2. I had a hard time understanding the legends of Figs 2D, 3C, 4C, and5C. For instance, in Fig. 2D, if I understood correctly, the blue circles correspond to an average of 10% of the RAB11-positive VLCs present in a given zone. There is a total of 8 blue circles. Should not it be 10 (100%). Also, only symbols that are used in the corresponding Fig. should be shown.

We are sorry for the confusing presentation of our data and implemented changes based on the reviewer's comments. We modified the legends in an attempt to make it clearer and we put the Figs 3C, 4C and 5C in supplementary data (Supp Fig. S2). The legends have been adapted, only what is shown is described in the legends. Note that the percentages of vacuoles in a specific location are only mentioned if they reach each threshold (10% or above). Because of this, the total percentage of vacuoles do not necessarily add up to 100% in the diagram.

3. The authors found surprising that some RAB6-positive VLCs are also labelled with RAB11. However, functional links between RAB6 and RAB11 have been already described (Miserey-Lenkei et al, Traffic 2007, 8 :1385).

We are thankful to the reviewer to point out important literature we missed. We changed the text and included the missing citation.

Reviewer 2:
1. With reference to the introduction it is adequate; it has all the relevant information as per the demand of the experiments carried out. However, some more knowledge about the different Rab proteins playing specific roles in epithelia other than Accessory Gland epithelium would be useful.

We have included a section in the introduction that highlights known Rab6, 7, 11, 19 specific functions within the context of secretory gland cells, such as salivary gland cells.

2. The results section is fine, however some important points have not been answered: From line no.124 onwards experiments regarding analysis of the morphology of cells of AGs have been analyzed using different polarity markers. Here, dE-Cadherin has been used to observe the apical domains of cells, however, Cadherins are basically cell-cell adhesion molecules, so what about the typical markers of the apical domains of the epithelial cells like crumbs or bazooka? Were they also checked? A detailed analysis of these complexes would be helpful to understand the morphology of the cells better.

Please refer to our response above. In short, the endogenous knock-in line expressing Crb-GFP and Dlg-RFP recapitulate our findings using DE-Cadherin (DCAD) (Fig. S12 and reviewers' Fig. R2).

3. References are not in the standard format. All the references should follow same pattern. No issue date or month and no issue number is given in standard reference writing.

We are sorry for this technical oversight. All references have been re-formatted.

Decision and Reviews

Dear Dr. Maeda,

Thank you for resubmitting your manuscript entitled "Rab-mediated trafficking in the secondary cells of Drosophila accessory glands" to Traffic. I asked both of the original referees to comment on the revised manuscript. Referee 1, whose brief comment is attached, is satisfied that you have addressed all of the previously raised concerns. Although Referee 2 has not yet responded, the original concerns of this referee were considered minor and it seems to me that you have adequately addressed them. I am therefore pleased to accept this paper for publication in Traffic. Congratulations on a nice piece of work, and thank you so much for considering Traffic as a venue for its publication!

Sincerely,

Michael S. Marks, Ph.D.
Co-Editor

Referee's Comments to the Authors

Referee: 1

Comments to the Author
The authors have met my previous comments in a satisfactory way.
